# Mutations Affecting Potassium Import Restore the Viability of the *Escherichia coli* DNA Polymerase III *holD* Mutant

**DOI:** 10.1371/journal.pgen.1006114

**Published:** 2016-06-09

**Authors:** Adeline Durand, Anurag Kumar Sinha, Cloelia Dard-Dascot, Bénédicte Michel

**Affiliations:** 1 Genome biology department, Institute for Integrative Biology of the Cell (I2BC), CEA, CNRS, Université Paris-Sud, Université Paris-Saclay, 91198 Gif-sur-Yvette, France; 2 High-throughput Sequencing facility, Institute for Integrative Biology of the Cell (I2BC), CEA, CNRS, Université Paris-Sud, Université Paris-Saclay, 91198 Gif-sur-Yvette, France; Université Paris Descartes, INSERM U1001, FRANCE

## Abstract

Mutants lacking the ψ (HolD) subunit of the *Escherichia coli* DNA Polymerase III holoenzyme (Pol III HE) have poor viability, but a residual growth allows the isolation of spontaneous suppressor mutations that restore Δ*holD* mutant viability. Here we describe the isolation and characterization of two suppressor mutations in the *trkA* and *trkE* genes, involved in the main *E*. *coli* potassium import system. Viability of Δ*holD trk* mutants is abolished on media with low or high K^+^ concentrations, where alternative K^+^ import systems are activated, and is restored on low K^+^ concentrations by the inactivation of the alternative Kdp system. These findings show that the Δ*holD* mutant is rescued by a decrease in K^+^ import. The effect of *trk* inactivation is additive with the previously identified Δ*holD* suppressor mutation *lexAind* that blocks the SOS response indicating an SOS-independent mechanism of suppression. Accordingly, although lagging-strand synthesis is still perturbed in *holD trkA* mutants, the *trkA* mutation allows HolD-less Pol III HE to resist increased levels of the SOS-induced bypass polymerase DinB. *trk* inactivation is also partially additive with an *ssb* gene duplication, proposed to stabilize HolD-less Pol III HE by a modification of the single-stranded DNA binding protein (SSB) binding mode. We propose that lowering the intracellular K^+^ concentration stabilizes HolD-less Pol III HE on DNA by increasing electrostatic interactions between Pol III HE subunits, or between Pol III and DNA, directly or through a modification of the SSB binding mode; these three modes of action are not exclusive and could be additive. To our knowledge, the *holD* mutant provides the first example of an essential protein-DNA interaction that strongly depends on K^+^ import *in vivo*.

## Author Summary

Replication polymerases are responsible for genome duplication; they are ubiquitous and show high levels of functional and structural conservation across all species. The HolC-HolD (χψ) complex is a component of the replicative polymerase in the model bacteria *Escherichia coli*, and is crucial for normal growth. We isolated suppressor mutations that restore the viability of the *holD* mutant and we found that inactivating the Trk system, responsible for the main pathway of potassium import, renders the entire χψ complex dispensable for growth. Activation of alternative pathways of potassium import abolishes the suppression. The viability of the *holD trk* mutant is due in large part to a better capacity of the χψ-less polymerase to compete with other polymerases. Potassium glutamate is the major intracellular ionic osmolyte in *E*. *coli*, and we propose that mutations that affect potassium concentration *in vivo* stabilize the χψ-less polymerase by increasing electrostatic interactions between the different polymerase subunits and between polymerase and DNA. Stabilized by the lower intracellular potassium concentration, the χψ-less polymerase becomes functional in *trk* mutants, to a level that permits cell growth with this defective polymerase. Our results imply that K^+^ import can play an important role in the stability of protein complexes on DNA.

## Introduction

Chromosome replication is performed in *Escherichia coli* by a replicase called the DNA Polymerase III holoenzyme (Pol III HE) and composed of 9 different polypeptides [1]. DNA synthesis is realized by the core polymerase, composed of a polymerase subunit (α, encoded by *dnaE*), associated with a proof-reading activity (ε, encoded by *dnaQ*) and a stabilizing subunit (HolE). Each Pol III HE contains two core polymerases, one for the continuously synthesized leading-strand, one for the lagging-strand synthesized as 1–2 kilobase (kb) Okazaki fragments. The presence of an additional spare one to form a trimeric polymerase was proposed and is still debated [2–4]. The lagging-strand template is transiently single-stranded during Okazaki fragment synthesis and covered by single-stranded DNA binding proteins (SSB). The stability of each core polymerase on DNA is ensured by its interaction with a polymerase clamp, in *E*. *coli* this is a β-dimer (encoded by *dnaN*) which encircles the DNA and is structurally homologous to PCNA in eukaryotes [5]. The β-clamp is loaded onto DNA by a clamp loader complex, functionally homologous to the RFC complex in eukaryotes, which, in addition to loading the β-clamp, ensures the cohesion of the replisome by interacting with the three core polymerases and with the DNA helicase. In *E*. *coli* the clamp loader complex is composed of three τ subunits (encoded by *dnaX*), the δ and δ’ subunits (encoded by *holA* and *holB*, respectively), and a heterodimeric complex of χ and ψ proteins, encoded by *holC* and *holD* respectively [1, 2]. The ψχ complex forms a bridge between the τ_3_δδ’pentamer and SSB. Actually, ψ interacts with τ and χ, which itself interacts with SSB [6–9]. In addition, the ψ-τ interaction favors the assembly of the τ_3_δδ’pentamer at limiting δδ’concentrations, stabilizes an ATP-activated DNA-high affinity conformation of the clamp loader, and thus facilitates the clamp loading reaction *in vitro* [10–13].

Most Pol III HE subunits are essential for growth, with the notable exception of HolE and DnaQ [14]. Inactivating *holC* is not lethal but impairs growth, particularly at a high temperature; growth of *holC* mutants can be significantly improved if the induction of the SOS response, a set of repair genes induced by DNA damage or replication impairment, is prevented [15, 16]. Inactivating *holD* is strongly deleterious in all growth conditions; however, a residual growth on minimal medium at 30°C facilitates the selection of suppressor mutations. We previously reported that Δ*holD* mutant growth is improved by mutations that inactivate the SOS response and more specifically by the inactivation of *dinB* and *polB* genes, encoding the SOS-induced bypass polymerases Pol IV and Pol II, respectively [17]. We proposed that increased (SOS-induced) levels of these polymerases compete with HolD-less Pol III HE and destabilize its interaction with DNA. The viability of *holD* and *holC* mutants is also restored by a duplication of the *ssb* gene, which doubles the intra-cellular level of SSB proteins [16]. We proposed that increasing intracellular concentration of SSB favors the SSB-DNA binding mode where each SSB tetramer binds 35 nucleotides, which stabilizes Pol III bound to DNA and bypasses the need for HolD. In the present study, we describe the isolation of *holD* suppressor mutations that affect K^+^ import.

K^+^ is the major intracellular cation in *E*. *coli*, present at concentrations that vary from 100–150 to 500–600 mM, which is higher than that in the extracellular medium [18–20]. In the growth medium used here, about 45–50% of K^+^ ions are expected to be “free” and balance charges of small diffusible anions, including glutamate, and about 50–55% are thought to be “bound” and serve to balance charge on macromolecule anions, proteins and nucleic acids [21]. K^+^ glutamate is the intracellular ionic compound that ensures turgor and osmolarity. K^+^ is imported in *E*. *coli* by several independent systems [22, 23], [24] (Table 1). The major one is composed of TrkA, TrkH or TrkG, and TrkE [22, 25]. These four genes are unlinked and represent two separate pathways, TrkAEG and TrkAEH. A second system depends on one protein, TrkD later called Kup [22, 26]. Trk proteins and Kup have a low affinity for K^+^ (Km 1.5 mM), and Trk is the main active pathway when K^+^ concentration in the growth medium is 5 mM or more; Kup activity could only be studied in mutants lacking the other K^+^ import systems [27, 28]. At low K^+^ concentrations the *kdpFABC* operon is induced by a two component system, KdpD and KdpE; Kdp is a high affinity K^+^ import system (Km 2 μM), active below 5 mM K^+^ [29, 30], [31]. Finally, at high K^+^ concentrations the triple *trk kup kdp* mutant imports K^+^ with a system called TrkF, which is a combination of several minor nonspecific (called “illicit”) transport pathways [32].

**Table 1:** K^+^ uptake systems in *E*. *coli*. (Adapted from [21] and [28]).

Name	Genetics	K_m_ (mM) / V_max_ (μmol.g^-1^ min^-1^)	Other properties
Kdp	6 genes, 2 operons *kdpFABC* encodes the structural proteins *kdpDE* encodes the regulatory proteins	0.002 / 100–150	*kdp FABC* operon is regulated by the KdpD, sensor kinase and the KdpE response regulator. It is expressed when the growth rate of cells begins to become limited by K^+^ availability
Trk	Four unlinked genes, *trkA*, *trkE* (also called *sapD*), *trkG* and *trkH*	1.5 / 300–500	Constitutively expressed; Corresponds to two pathways: TrkAEG and TrkAEH.
Kup	*kup* (also called *trkD*)	0.5 / 0–50	Transports Cs as well as K^+^
TrkF	Multiple “aberrant” K^+^ transport activities		Allows the *kdp trk kup* triple mutant to grow at high K^+^ concentrations (above 100 mM)

In this work, we show that *trkA* and *trkE* mutations restore the Δ*holD* mutant growth. The viability of Δ*holD* Δ*trk* mutants requires the Kdp and the TrkF K^+^ import pathways to be of low or negligible activity. We propose that HolD-less Pol III HE can replicate *E*. *coli* chromosomes when K^+^ intracellular concentration is affected, owing to its stabilization on DNA by improved electrostatic interactions.

## Results

### Isolation of Δ*holD* suppressor mutations

As inactivation of the *holD* gene prevents *E*. *coli* growth, Δ*holD* mutants were constructed and propagated in the presence of pAM-holD, a plasmid that carries the *holD* wild-type gene and replicates only in the presence of IPTG. Suppressors of the Δ*holD* growth defects can be obtained by growing Δ*holD* [pAM-*holD*] cells in the absence of IPTG and selecting for plasmid-less fast growing clones [16, 17]. Four such Δ*holD* fast growing colonies were isolated on minimum medium (M9) at 37°C in an MG1655 background (JJC6376 to JJC6379) (unless otherwise indicated, all minimal media used in this work contain 0.4% glucose and 0.2% casamino acids). The SOS response is induced in the Δ*holD* mutant and a *sfiA* mutant was used to prevent cell division blockage by the SOS-induced SfiA protein. Suppressor mutations were also isolated in a Δ*holD sfiA*::Mu strain, and two fast growing colonies obtained on M9 at 30°C were studied (JJC6389 and JJC6390). Interestingly, whole genome sequencing identified the presence of a mutation affecting the *trk* pathway of K^+^ import in two of these six clones. These mutations were confirmed by re-sequencing the genes of interest. JJC6377 carries a 84 base pair (bp) in-frame deletion in the *trkA* gene, from nucleotides 513 to 597, with 9 bp microhomology at the junction (called hereafter Δ*trkA*^84^, S1 Fig). It also carries a large duplication, from about 3 648 000 to around 4 167 000. It has to be noted that a *holD* mutation was shown to increase the frequency of recombination events between repeated sequences, which may account for the recurrent presence of deletions and duplications in suppressed *holD* mutants [16, 33]., JJC6389 carries a *trkE* point mutation at position 767 changing glutamine 255 to proline. This was the only sequence modification identified in this strain. The suppressor mutations identified in the four other sequenced genomes will be described in future publications.

### The Δ*trkA*^84^ deletion, a Δ*trkA* or Δ*trkE* full gene deletion is sufficient for suppression of the Δ*holD* growth defects

**Fig 1:**
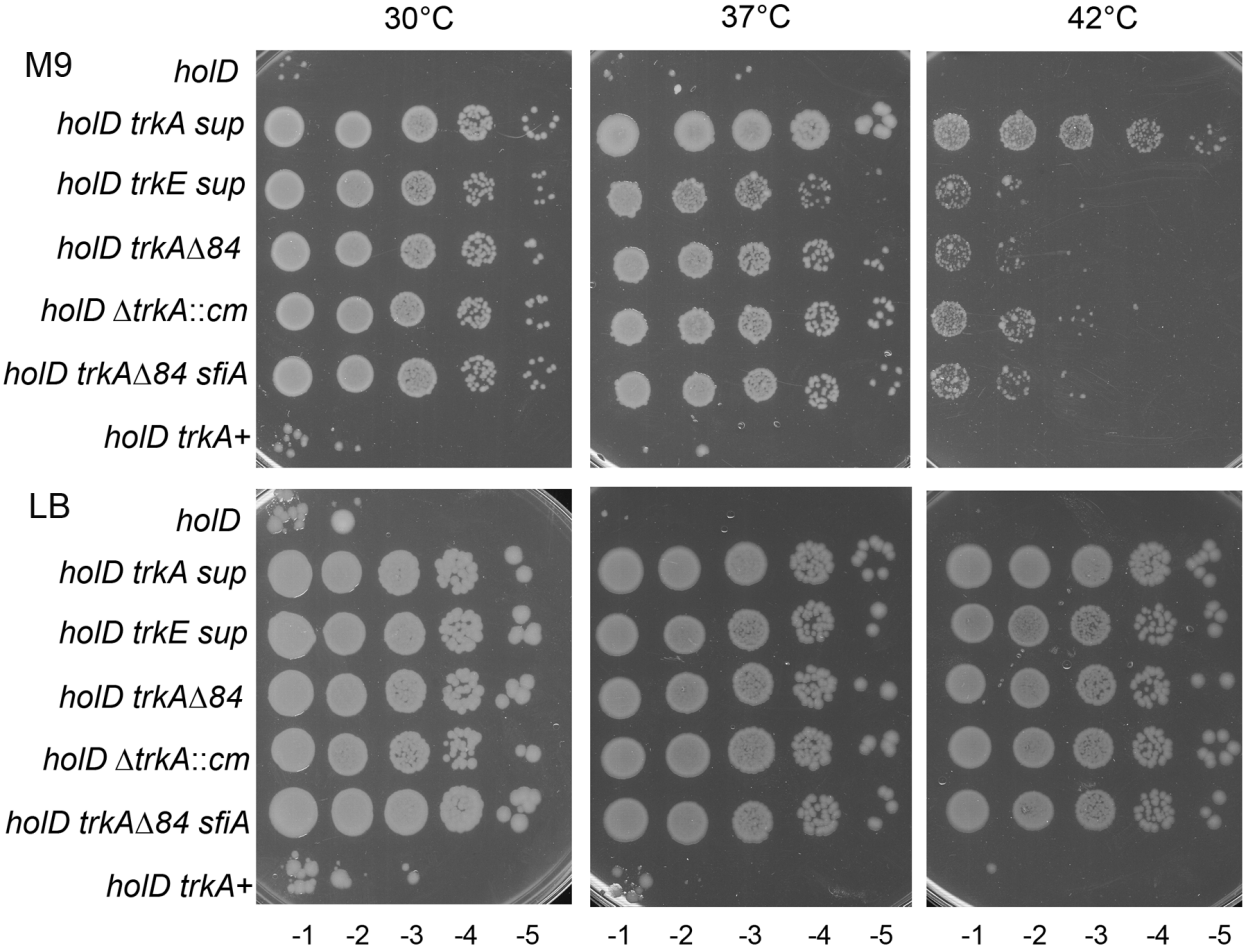
*trkA* and *trkE* mutations restore Δ*holD* viability. In a first step pAM-holD containing cultures were grown for 8 hours in M9 in the absence of IPTG at 30°C, appropriate dilutions were plated on M9 and plates were incubated for 3 days at 30°C. Isolated colonies, cured of pAM-holD, were suspended in 1 ml M9 salt medium and 5 μl drops of serial dilutions were plated on M9 or LB. LB plates were incubated overnight at 42°C or 37°C, or two days at 30°C. M9 plates were incubated for two days at the indicated temperatures. Isolated colonies obtained by streaking *holD trkA sup*, JJC6377 and *holD trkE sup*, JJC6389 on M9 at 30°C were tested in parallel. The strains used to generate plasmid-less colonies are: *holD*, JJC6869; *holD* Δ*trkA*^84^, JJC6669; *holD* Δ*trkA*::cm, JJC6682; *holD* Δ*trkA*^84^ *sfiA*, JJC6969; *holD trkA*^+^, JJC6670; *holD* Δ*trkE*::kan JJC7173.

The two Δ*holD* clones affected for K^+^ import formed colonies on LB at 30°C, 37°C, and 42°C overnight. On M9, the strain carrying Δ*trkA*^84^ formed colonies at all temperatures, while the strain mutated in *trkE* formed colonies at 30°C and 37°C only (Fig 1, the two original suppressed clones are called Δ*holD trkA sup* and Δ*holD trkE sup*). However, the spontaneous suppressor strain carrying Δ*trkA*^84^ also carries a large duplication in addition to the Δ*trkA*^84^ mutation, which could improve its viability, whereas the *trkE*^Q255P^ mutation was the sole genome modification identified in JJC6389. Co-transduction of Δ*trkA*^84^ with an adjacent marker (*zhd-3082*::Tn*10*) in a Δ*holD* [pAM-holD] or in a Δ*holD sfiA* [pAM-holD] background yielded clones that, after plasmid loss, were able to grow on LB at all temperatures and on M9 at 30°C and 37°C, and were only slightly more viable than the *trkE*^Q255P^ suppressed strain at 37°C (Fig 1 Δ*holD* Δ*trkA*^*84*^, Δ*holD* Δ*trkA*^*84*^ *sfiA*). A Δ*trkA*::Cm mutant lacking the entire *trkA* sequence was constructed by gene replacement and behaved as the Δ*trkA*^84^ deletion (Fig 1 Δ*holD trkA*::Cm), suggesting that the Δ*trkA*^84^ deletion is a null allele. The use of a null allele of *trkE* showed that the inactivation of *trkE* or *trkA* restores *holD* mutant growth to the same extent, as expected since these two genes belong to the same pathway. The *trkE*^Q255P^ mutation was slightly less efficient than the Δ*trkE* deletion in restoring *holD* mutant growth at 37°C, suggesting a residual activity of the mutant protein. We conclude that inactivation of the *trk* pathway of K^+^ import restores growth of the Δ*holD* mutant on LB at all temperatures and on M9 at 30°C and 37°C.

### Rescue of Δ*holD* growth by *trk* inactivation is abolished at low K^+^ concentrations in a *kdp*-dependent way

**Fig 2:**
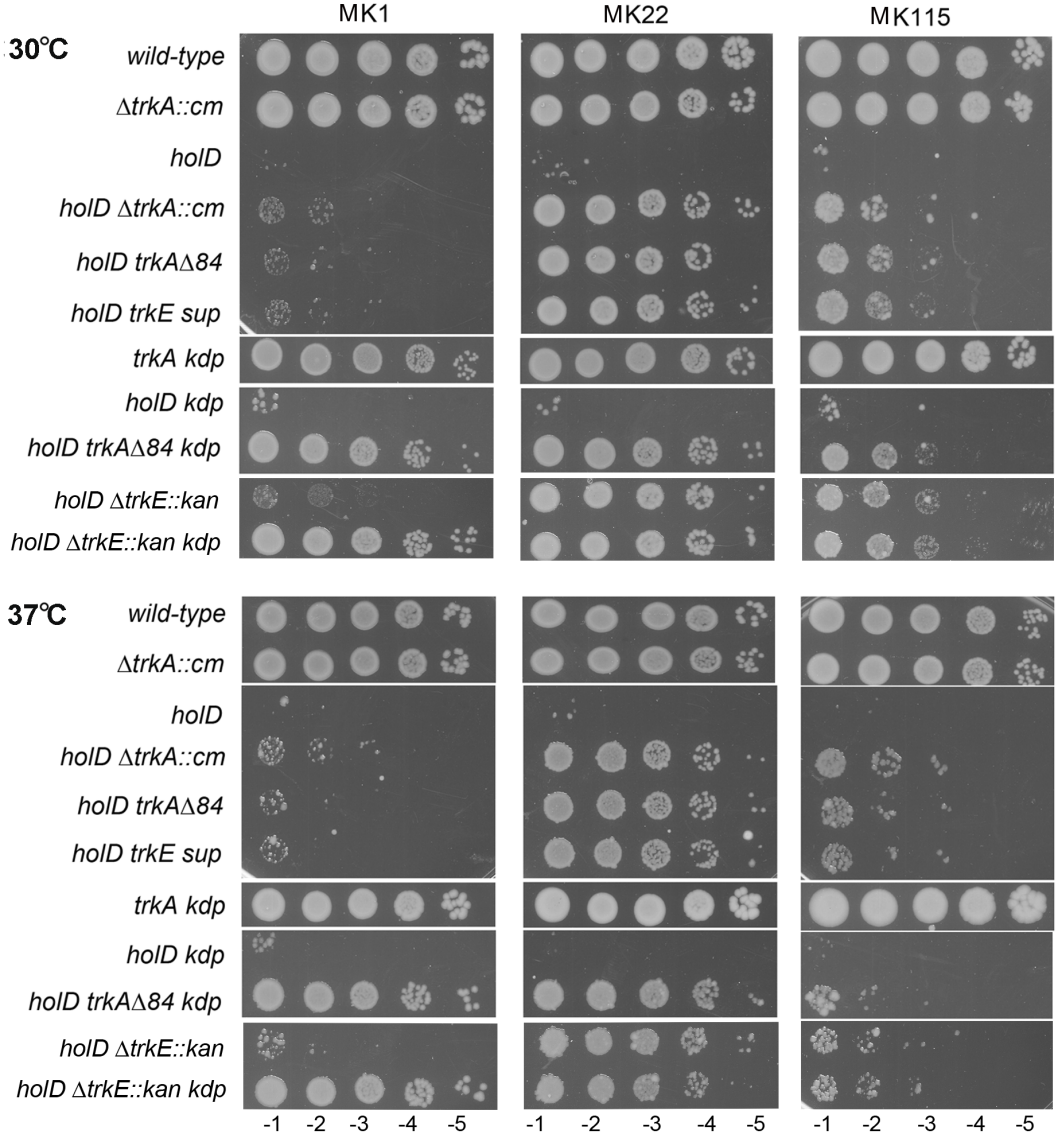
Viability of Δ*holD trkA* and Δ*holD trkE* mutants is abolished by a high K^+^ concentration, and in a Kdp-dependent way at a low K^+^ concentration. In a first step, isolated colonies were obtained by plating appropriate dilutions of wild-type, Δ*trkA*, *holD trkE sup* or Δ*trkA* Δ*kdp* overnight cultures on M9, or appropriate dilutions of pAM-holD containing cultures grown for 8 hours in the absence of IPTG at 30°C, and incubating plates for 3 days at 30°C. These isolated colonies were suspended in 1 ml MK0 salt medium. Serial dilutions were made and 5 μl drops of each dilution were plated on MK plates containing different K^+^ concentrations as indicated (MK1 1 mM, MK22 22 mM, MK115 115 mM; see S2 Fig for growth on MK0.2, M9, MMA, and LB). Pictures of wild-type and Δ*trkA* strains at 37°C were taken after 24 h incubation; all other plates were incubated for two days at the indicated temperatures. Wild-type, JJC1392; Δ*trkA*::cm, JJC6800; *holD trkE sup*, JJC6389 (the original suppressed clone); Δ*trkA*^84^ Δ*kdp*::cm, JJC7030. The strains used to generate plasmid-less colonies are: *holD*, JJC6869; *holD* Δ*trkA*::cm, JJC6898; *holD* Δ*trkA*^84^, JJC6969; *holD* Δ*kdp*::cm JJC6750; *holD* Δ*trkA*^84^ Δ*kdp*::cm, JJC6819; *holD* Δ*trkE*::*kan*, JJC7173; *holD* Δ*trkE*::kan Δ*kdp*::cm, JJC7223.

To check that the Δ*trkA*^84^, Δ*trkA*::Cm, Δ*trkE*::*kan* and *trkE*^Q255P^ mutations allow Δ*holD* mutant growth by affecting K^+^ import, we tested the effect of different K^+^ concentrations in the external medium on the viability of the different Δ*holD trk* mutants. In K^+^-limiting conditions the *kdpFABC* operon is induced by the regulatory proteins KdpD and KdpE [30]. As shown in Figs 2 and S2, Δ*holD* Δ*trkA*^84^, Δ*holD* Δ*trkE* and Δ*holD trkE*^Q255P^ did not form colonies on minimal medium containing 0.2 mM or 1 mM K^+^ (called MK0.2 and MK1, respectively), although, as expected, they could grow on the same medium containing 22 mM K^+^ (MK22), the K^+^ concentration in M9. Lethality of Δ*holD trk* mutants on plates containing low K^+^ concentrations may result from the activation of the *kdp* operon, which promotes active K^+^ uptake below 5 mM in the external medium [30]. Inactivation of *kdpA* restored the growth of Δ*holD* Δ*trkA*^84^ and Δ*holD* Δ*trkE* mutants on MK0.2 and MK1 (Δ*holD* Δ*trkA*^84^ Δ*kdp*, Δ*holD* Δ*trkE* Δ*kdp*, Figs 2 and S2), indicating that the lethality of *holD trk* mutants at low K^+^ concentrations results from the activity of Kdp. However, inactivation of *kdp* in a Trk^+^ Δ*holD* did not restore viability at low K^+^ concentrations, with the exception of a partial growth at 30°C on MK0.2 (Δ*holD* Δ*kdp* Figs 2 and S2). Because the Trk system is constitutively expressed and has a high V_max_ it is still partly active even on MK0.2 (the growth rate of the *kdp* mutant is only 20% lower in 0.2 mM than in 5 mM K^+^ [18, 34]). On MK0.2, the remaining activity of Trk in the *holD kdp* mutant is responsible for the growth defect, but this activity is limited, allowing a weak but significant residual growth at 30°C. Finally, it has to be noted that the K^+^ concentration in our LB was measured and found to be 10.9 ± 0.74 mM, but as shown below other components than K^+^ also play a role in the growth of Δ*holD* Δ*trk* mutants on LB.

The Trk system is the main K^+^ import system in *E*. *coli*. Therefore, inactivating *trkA* might decrease K^+^ intracellular concentration in growing cells. As shown in Table 2, intracellular K^+^ concentration was significantly decreased in *trkA* and *holD trkA* mutants compared to wild-type cells. This result suggests that a 12–17% decrease in intracellular K^+^ is sufficient to promote HolD-less Pol III stabilization on DNA.

**Table 2:** *trkA* gene inactivation decreases *E*. *coli* intracellular K^+^ concentration.

Strain	Genotype	nmoles K^+^/mg DW	Ratio to wt	N
JJC1392	wild-type	502 ± 12	1	8
JJC6800	*trkA*	443 ± 12	0.88	7
JJC6898S	*holD trkA*	418 ± 10	0.83	7

Cells grown to OD 0.4 in M9 were washed in hypertonic, potassium-free glucose medium and dried. K^+^ concentrations were measured in dried pellets with a flame spectrophotometer (see Material and Methods for details). Results are expressed in nmoles K^+^ per milligram of dry weight (DW). Averages of N independent experiments ± Standard Error of the Mean (SEM) are shown. Results were compared by a Tukey-Test (multiple comparisons) in conjunction with an ANOVA: *trkA* and *holD trkA* mutants were both significantly different from wild-type (P = 0.004 and P = 0.00014, respectively) and were not significantly different from each other (P = 0.307). (“JJC6898S” stands for “segregated” cultures).

In conclusion, when Δ*holD trkA* (or *holD trkE*) cells are grown on 22 mM K^+^ (M9 or MK22), K^+^ uptake is impaired and intracellular K^+^ concentration decreases to a level that rescues the Δ*holD* mutant. At low K^+^ concentrations (0.2 mM and 1 mM) the activation of the *kdp* operon increases K^+^ uptake to a level that prevents the growth of Δ*holD* Δ*trkA* and Δ*holD* Δ*trkE* mutants. By preventing Kdp-mediated K^+^ import, *kdp* gene inactivation restores growth of the Δ*holD* Δ*trkA* Δ*kdp* and Δ*holD* Δ*trkE* Δ*kdp* mutants. We conclude that viability of the Δ*holD* mutant can be restored by decreasing K^+^ import.

### Growth of the Δ*holD trk* mutants is prevented by a high K^+^ concentration and unaffected by *kup* inactivation

Mutants lacking all three K^+^ import systems (*trk*, *kdp*, *kup*) require a high K^+^ concentration for growth and rely on multiple minor K^+^ import activities called TrkF [32]. To test whether TrkF activity prevents growth of the *holD trkA* and *holD trkE* mutants on medium containing a high level of K^+^, colony formation of the Δ*trkA*, Δ*holD* Δ*trkA*, Δ*holD* Δ*trkE* and Δ*holD trkE*^Q255P^ mutants was compared on synthetic medium containing 22 mM (M9, MK22), 115 mM (MK115) or 150 mM (MMA) K^+^. As shown in Figs 2 and S2, a high concentration of K^+^ prevented growth of the Δ*holD trk* mutants; therefore activating K^+^ import by a high concentration of K^+^ in the growth medium is lethal to the Δ*holD trk* mutants. It was not possible to inactivate TrkF, which is not a defined locus but a combination of several minor pathways [32].

**Fig 3:**
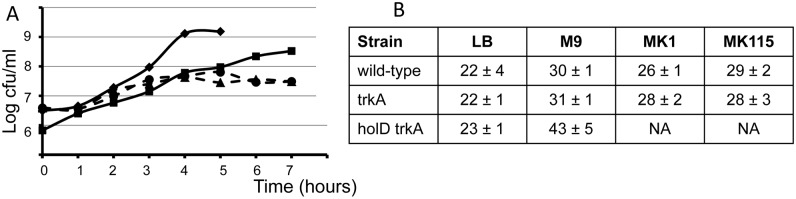
Viability of the *holD trkA* mutant in liquid medium. Overnight cultures of wild-type (JJC1392), *trkA* (JJC6800) and *holD trkA* [pAM-holD] (JJC6898) strains were grown at 30°C in LB, M9, MK1, MK115 medium, diluted, and further grown at 37° in the same medium for 7 hours, as described in the Materials and Methods. (A) Representative growth curves of *holD trkA* (JJC6898) in LB (full line, diamond), M9 (full line, square), MK1 (dashed line, circles), MK115 (dashed line, triangles). (B) The doubling time expressed in minutes was calculated from the exponential part of the growth curve, averages of three independent determinations are shown with standard deviations. No generation time could be calculated for the *holD trkA* mutant grown in MK115 and in MK1, as growth at 37°C stopped after 2 to 3 generations (NA = not applicable).

Growth of the Δ*holD* Δ*trkA* mutant was compared in different liquid media (Fig 3). For these experiments, the Δ*holD* Δ*trkA* [pAM-holD] mutant was grown overnight at 30°C to saturation in LB, M9, MK1 or MK115 medium without IPTG, diluted to OD 0.002 in the same medium, and grown at 37°C; growth was monitored by plating appropriate dilutions on M9. As shown in Fig 3A, growth was rapid in LB, slower in M9, and stopped after two or three generations in MK1 and MK115, in agreement with the lack of colony formation at these K^+^ concentrations. Generation times were calculated from the slope of the best fit straight line during exponential growth (Fig 3B, generation times could not be calculated for the Δ*holD* Δ*trkA* mutant grown in MK1 or MK115 owing to the rapid growth arrest). The growth rate of the single Δ*trkA* mutant was similar in M9, MK1 and MK115, and not significantly different from wild-type (30 min), while the generation time of Δ*holD* Δ*trkA* cells in M9 was nearly 50% longer (43 min). Surprisingly, the generation time of Δ*holD* Δ*trkA* cells was similar to that of wild-type and Δ*trkA* single mutant in LB (22–23 min), in agreement with overnight colony formation on LB, confirming that the rescue of the *holD* mutant by *trkA* is very efficient in LB.

The *kup* gene, originally called *trkD*, is constitutively expressed and active in the same growth conditions as the Trk system, but Kup has a low level of activity [22, 26]. A Δ*kup* mutant was used to test whether the Kup pathway plays a role in the viability of Δ*holD* and Δ*holD* Δ*trkA* mutants at different K^+^ concentrations. Inactivating *kup* did not rescue the Δ*holD* mutant on M9 (S3A Fig), in agreement with the idea that Trk is the main K^+^ import pathway under these conditions. It did not affect the growth of the Δ*holD* Δ*trkA* mutant at any K^+^ concentration tested (S3A Fig), including in 10.9 mM K^+^, the K^+^ concentration in our LB. These results are in agreement with the idea that Trk is the major K^+^ import system on M9, and that the Kdp K^+^ import system is activated at low K^+^ concentrations. They also suggest that at high K^+^ concentrations the poor viability of Δ*holD trkA* cells is caused by TrkF activity, since Kup and TrkF are the only active pathways at high K^+^ concentrations in a *trk* mutant, and the phenotype of the *holD trk* mutant is not affected by Kup inactivation.

### All LB components participate to the *holD trkA* mutants growth on LB at 42°C

**Fig 4:**
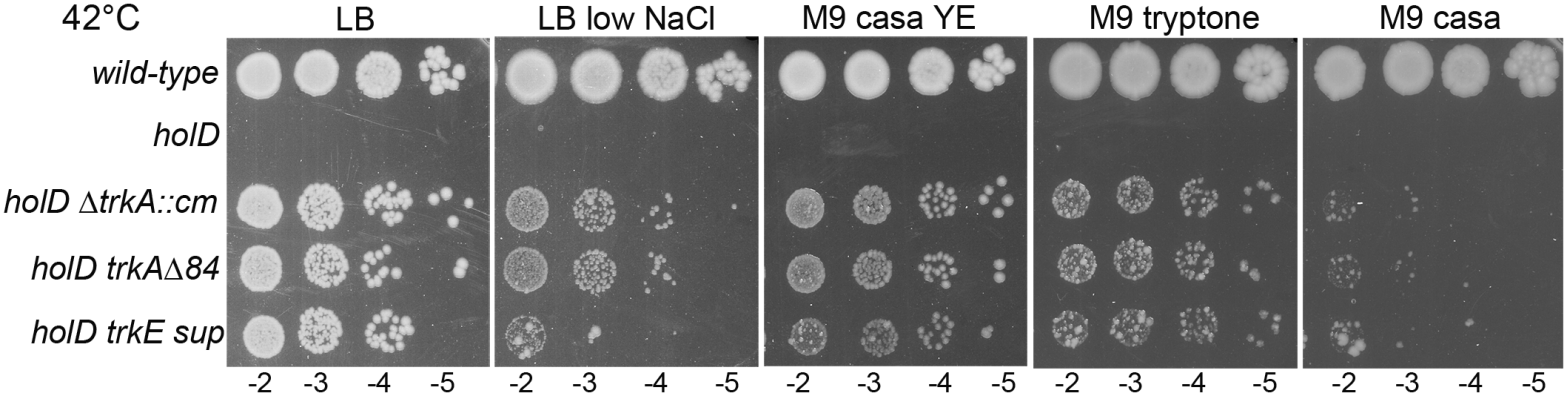
All LB components participate to the viability of *holD trkA* mutants at 42°C. Isolated colonies obtained on M9 as described in the legend of Fig 2 were suspended in MK0 salts, and 5 μl drops of serial dilutions were plated on the indicated medium. LB and M9 casamino acids yeast extract plates were incubated overnight. M9 casamino acids and M9 tryptone plates were incubated for two days at 42°C. Wild-type, JJC1392; *holD trkE sup*, JJC6389. The strains used to generate plasmid-less colonies are: *holD*, JJC6869; *holD* Δ*trkA*::*cm*, JJC6898; *holD* Δ*trkA*^84^, JJC6969.

Surprisingly, at 42°C Δ*holD trkA* mutants formed colonies overnight (ON) on LB but not on minimal medium (Fig 1). This result was unexpected since the number of replication forks per cell is increased in rich medium compared to minimal medium, which is expected to disfavor a mutant that lacks a Pol III HE subunit. Furthermore, LB contains 10.9 mM K^+^, a concentration that does not allow growth of the Δ*holD trkA* mutant at 42°C in synthetic medium (S3A Fig). Consistent with the idea that a low intracellular K^+^ concentration restores viability by stabilizing HolD-less Pol III HE-DNA complexes, the lethality on M9 at 42°C could result from a destabilization of replication complexes by temperature. To understand why this destabilization is not observed in LB, we tested the three LB components individually (tryptone, yeast extract and NaCl). As shown on Fig 4, adding yeast extract to M9 casamino acids medium allowed growth of the Δ*holD* Δ*trkA* and Δ*holD trkE*^Q255P^ mutants at 42°C. Tryptone is the product of casein hydrolysis by trypsin, while casamino acids are the product of acidic hydrolysis of casein. Consequently, tryptone contains mainly peptides while casamino acids contain mainly free amino acids. Replacing casamino acids by tryptone in M9 improved growth of *holD trk* mutants, although colonies were heterogeneous in size, possibly because growth remains slow, favoring the appearance of new suppressor mutations. Therefore, although yeast extract is more efficient than tryptone, each of these two components can improve growth at 42°C in M9. Note that the presence of casamino acids increases growth rate, but does not affect the plating efficiency of *holD trkA* cells at any temperature (S3B Fig).

The salts in M9 are 92.5 mM Na^+^ and 22 mM K^+^, while LB is 171 mM NaCl (10 g/l) and 10.9 mM K^+^. The Δ*holD* Δ*trkA* mutants formed slow growing colonies and Δ*holD trkE*^Q255P^ did not grow at 42°C on low salt LB (0.5 g/l NaCl, 8.5 mM) (Fig 4). As on M9 at 37°C, the Δ*holD trkE*^Q255P^ mutant was more impaired than the Δ*holD* Δ*trkA* mutant, presumably because of a residual activity of the mutated TrkE protein. Nevertheless, the high Na^+^ concentration in LB improves growth of both Δ*holD* Δ*trkA* and Δ*holD trkE*^Q255P^ mutants at 42°C. In conclusion, all three components of LB, and particularly yeast extract in the presence of high Na^+^, participate to the viability of the Δ*holD trk* mutants at 42°C.

### The Δ*trkA* mutation suppresses the growth defect of *holC* and *holC holD* mutants at 30°C and 37°C

**Fig 5:**
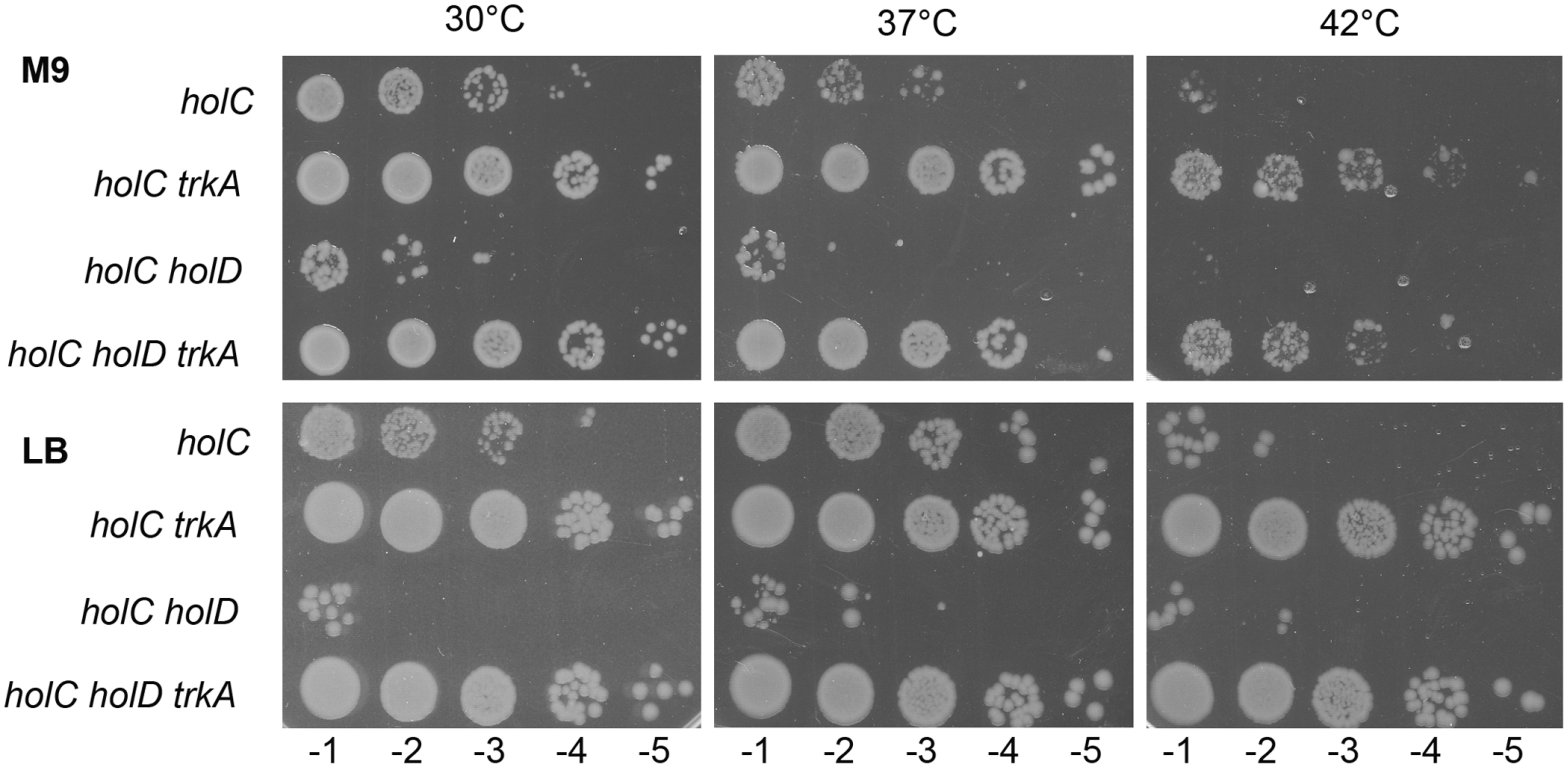
*trkA* inactivation restores Δ*holC* and Δ*holC* Δ*holD* viability. Isolated colonies obtained on M9 as described in the legend of Fig 2 were suspended in MK0 salts and 5 μl drops of serial dilutions were plated on the indicated medium (see S4 Fig for growth on MK1, MK22 and MK115). LB plates were incubated overnight at 37°C and 42°C, and for two days at 30°C. M9 casamino acids plates were incubated for two days at all temperatures. The strains used to generate plasmid-less colonies are: *holC*, JJC6748; *holC* Δ*trkA*^84^, JJC6827; *holC holD*, JJC6774*; holC holD* Δ*trkA*^84^, JJC6828.

ψ (HolD) plays a dual role in the clamp loader complex: its interaction with τ (DnaX) stabilizes the complex, and its interaction with χ (HolC) connects clamp loading and Okazaki fragment synthesis through the χ-SSB interaction. Accordingly, a Δ*holC* mutation, which lacks only the clamp loader-SSB interaction, is less deleterious than the Δ*holD* mutation, particularly at 30°C [16] (Figs 1 and 5). The Δ*trkA* mutation improved the Δ*holC* mutant growth at 30°C, 37°C, and 42°C on LB, and at 30°C and 37° on M9. Results were variable on M9 at 42°C, with either no growth of the Δ*holC* Δ*trkA* mutant or, as in the example shown in Fig 5, appearance of new suppressor mutations. Δ*holC* Δ*holD* Δ*trkA* mutant viability was similar to that of the Δ*holD* Δ*trkA* mutant, showing that suppression of the Δ*holD* growth defects by *trkA* inactivation does not require χ (Fig 5).

The effects of K^+^ concentration on the suppression of the *holC* mutant growth defects by *trk* inactivation were tested (S4 Fig). At 30°C, presumably owing to a significant growth of the Δ*holC* mutant, the Δ*trkA* mutation improved growth at all K^+^ concentrations, forming smaller colonies only on MK1. Therefore K^+^ import by Kdp or TrkF did not prevent Δ*holC* Δ*trkA* mutant growth. However, at 37°C where growth of the Δ*holC* mutant is severely impaired, rescue by the Δ*trkA* mutation was efficient only on 22 mM K^+^, while on low and high K^+^ concentrations colonies were highly heterogeneous, indicating that improvement of residual growth mainly favored the appearance of new suppressor mutations. As expected, rescue of Δ*holC* Δ*holD* mutant was weak or null at low or high K^+^ concentrations compared to rescue at 22 mM (S4 Fig). Therefore, a defect in K^+^ import allows chromosome replication in the absence of either ψ, or χ or both.

### *trkA* does not restore *holD* mutant growth by affecting SOS induction and does not restore a wild-type level of primer elongation

**Fig 6:**
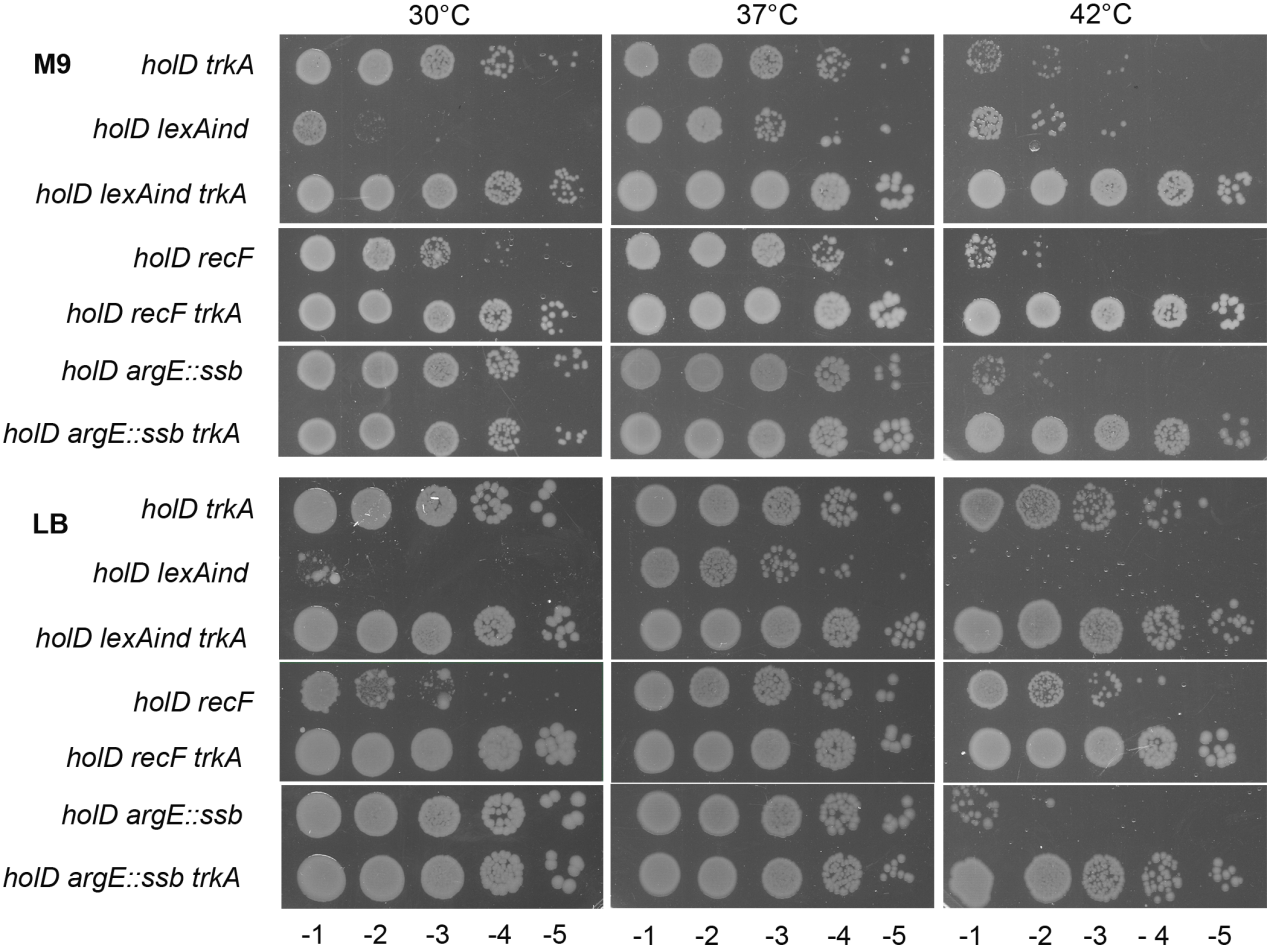
*trkA* inactivation does not rescue the Δ*holD* mutant by affecting SOS induction or SSB binding. Isolated colonies obtained on M9 as described in the legend of Fig 2 were suspended in MK0 salts and 5 μl drops of serial dilutions were plated on M9 and LB (see S5 Fig for growth on MK1, MK22, MK115). LB plates were incubated overnight at 37°C and 42°C, and for two days at 30°C. M9 casamino acids plates were incubated for two days at all temperatures. The strains used here to generate plasmid-less colonies are: *holD* Δ*trkA*::cm, JJC6898; *holD lexAind*, JJC6420; *holD lexAind* Δ*trkA*::cm, JJC7008; *holD recF*, JJC7058; *holD* Δ*trkA*::cm *recF*, JJC7063; *holD argE*::*ssb*, JJC6394; *holD argE*::*ssb* Δ*trkA*::cm, JJC7000.

We previously showed that growth of the Δ*holD* mutant is improved upon inactivation of SOS by a *lexAind* or a *recF* mutation [17]. These suppressor mutations were more efficient in the AB1157 context than in the context used here, MG1655, where Δ*holD lexAind* and Δ*holD recF* viability was mainly improved at 37°C ([16] (Fig 6). We tested whether the *trk* mutations act through a decrease of SOS induction. Measures of SOS induction using a *sfiA*::*lacZ* fusion showed that the SOS response was induced in the Δ*holD* Δ*trkA* mutant as in the Δ*holD* mutant (Table 3). SOS induction was RecF-dependent indicating that it results from the accumulation of single-stranded DNA gaps in the Δ*holD* Δ*trkA* mutant as in the Δ*holD* mutant. Furthermore, preventing SOS induction and *trk* inactivation showed an additive effect on the viability of the *holD* mutant, as Δ*holD* Δ*trkA lexAind* and Δ*holD* Δ*trkA recF* mutants were viable on M9 at 42°C (Fig 6). Rescue of the Δ*holD lexAind* and Δ*holD recF* mutants by Δ*trkA* was only efficient on 22 mM K^+^ and was not observed at low or high K^+^ concentrations, indicating that it requires alternative K^+^ import systems to be of low or negligible activity (S5 Fig). We conclude from these experiments that Δ*trkA* did not restore the viability of the Δ*holD* mutant by preventing RecF-dependent SOS induction, and that the Δ*holD* Δ*trkA* mutant still accumulates single-stranded DNA gaps during replication.

**Table 3:** The *holD trkA* mutant constitutively expresses the SOS response.

strain	relevant genotype	Β-gal Miller Units	N
JJC6478	Wild-type	35 ± 1.7	3
JJC6897	Δ*trkA*	33.5 ± 2	4
JJC6545S	Δ*holD*	168 ± 28	6
JJC6969S	Δ*holD* Δ*trkA*	228 ± 25	7
JJC7058S	Δ*holD recF*	81 ± 7	3
JJC7067	Δ*holD* Δ*trkA recF*	81 ± 9	3

Strains harboring a *lacZ* deletion and a *sfiA*::*lacZ* fusion were used to measure SOS constitutive expression in different mutants as described in Materials and Methods. JJC7067 was constructed by introduction of the *sfiA*::MudAp*lacZ* fusion in a Δ*holD* Δ*trkA recF* clone previously cured of pAM-holD. For JJC6545, JJC6969 and JJC7058, overnight cultures were grown in the absence of IPTG at 30°C in M9, diluted 50-fold in M9 at 30°C and grown to OD 0.2 to 0.6 for β-galactosidase tests (“S” stands for “segregated” cultures). Averages ± standard deviations are shown. N indicates the number of independent experiments.

If these gaps result from a delay in the use of RNA primers for the synthesis of Okazaki fragments, the strain might be sensitive to an excess of RNase H, which could destroy the RNA primers prior to their elongation. The results in Table 4 shows that the presence of a 20 copy plasmid that expresses RNase H prevented the formation of Δ*holD* Δ*trkA* colonies, although it did not affect the growth of HolD^+^ cells, suggesting that single-stranded DNA gaps are, at least in part, caused by a defect in RNA primer elongation.

**Table 4:** The *holD trkA* mutant is sensitive to RNaseH overexpression.

Strain	relevant genotype	% of plasmid-less colonies
JJC6669	Δ*holD* Δ*trkA*	85 ± 7.7
JJC6736	Δ*holD* Δ*trkA* [pACYC184]	91.4 ± 3
JJC6737	Δ*holD* Δ*trkA* [pEM001]	0.5
JJC7267	HolD^+^ Δ*trkA* [pACYC184]	91 ± 7
JJC7278	HolD^+^Δ*trkA* [pEM001]	97.6 ± 1

The indicated strains were propagated in M9 at 30°C for 8 hours for curing pAM-holD. The ratios of Spc^S^ colonies were calculated from the plating efficiency on M9 and M9 IPTG Spc. Averages and standard deviations of three independent experiments are shown. Colony forming efficiency on M9 IPTG Spc was not affected by the presence of pACYC184 or pEM001, and the number of colonies cured of pAM-holD on M9 was similar in the presence or absence of the vector pACYC184. A total of only five colonies cured of pAM-holD were obtained in three independent experiments in the presence of pEM001 in the *holD trkA* mutant (JJC6737), these colonies were highly heterogeneous upon streaking, indicating that pEM001 is highly deleterious for *holD trkA* cells.

**Fig 7:**
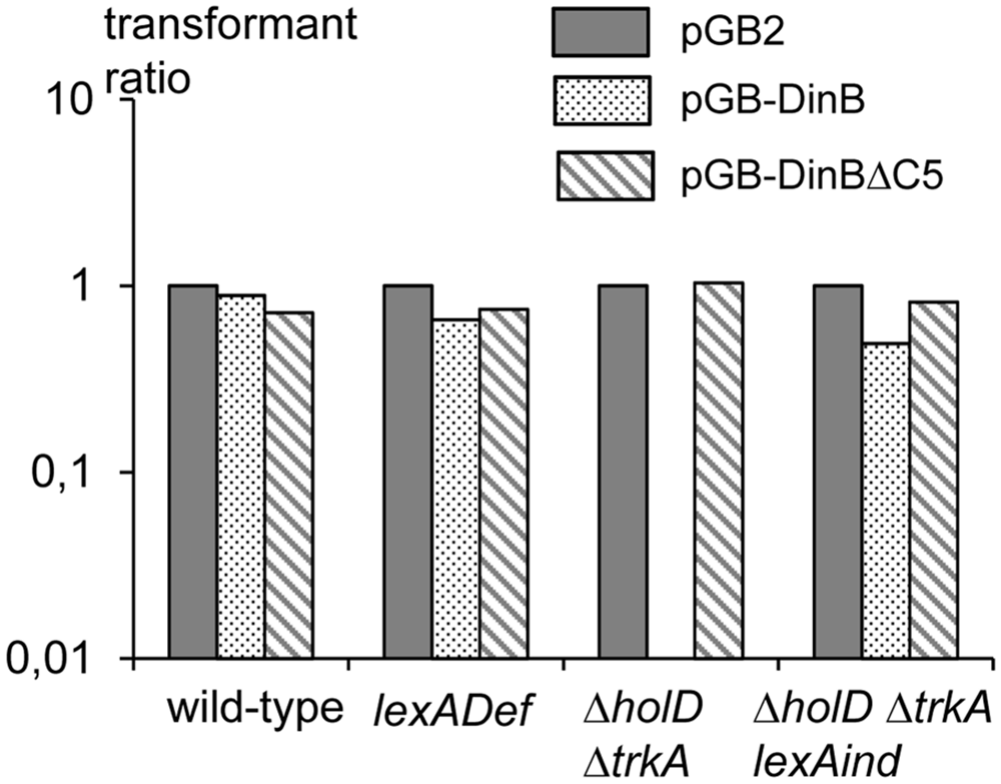
The *holD trkA* mutant is resistant to an increased level of DinB, but less than wild-type. Wild-type, (JJC6534), *lexADef* (JJC6904), *holD* Δ*trkA*::cm (JJC6927) and *holD* Δ*trkA*::*cm lexAind* (JJC7004) strains were transformed with plasmids pGB2 (plain bars), pGB-DinB (dotted bars) and pGB-DinBΔC5 (hatched bars). Transformants were selected on M9 spc plates. For each experiment, the number of pGB-DinB and pGB-DinBΔC5 transformants was normalized to the number of pGB2 transformants and the averages of three independent experiments are shown. Colonies obtained with pGB-DinB in the *holD trkA lexAind* mutant were smaller than pGB2 and pGB-DinBΔC5 colonies in the same strain, and than pGB-DinB colonies in wild-type and LexADef strains.

We previously showed that two SOS-induced proteins play an important role in the Δ*holD* mutant growth defects, the bypass polymerases DinB (Pol IV) and Pol II, and we proposed that these SOS-induced proteins are deleterious in the *holD* mutant because they compete efficiently with HolD-less Pol III for β-clamps binding at replication forks [17]. Accordingly, the over-production of DinB with a deletion of the last 5 amino acids (DinBΔC5), which fails to bind DnaN [35], was not lethal in a Δ*holD ssb*-duplicated strain [16]. The amount of DinB is expected to be similar in *holD trkA*, where DinB is 5- to 8-fold over-expressed owing to SOS-induction, and in *holD trkA lexAind* [pGB-DinB], where it is not SOS-induced but expressed from a 8–10 copy pSC101 vector [16, 36]. pGB2-DinB could be introduced by transformation in the Δ*holD* Δ*trkA lexAind* mutant, in which SOS is inactivated, (Fig 7), however it slightly slowed down growth, as cells harboring pGB2-DinB formed smaller colonies than those harboring the vector pGB2 or the control plasmid pGB-DinBΔC5. In the Δ*holD* Δ*trkA* mutant, where the SOS response is induced, pGB2 and pGB-DinBΔC5 could be introduced while pGB-DinB could not (Fig 7). We conclude that the Δ*trkA* mutation restores growth by preventing SOS-induced DinB proteins from destabilizing the HolD-less Pol III HE, but Δ*holD* Δ*trkA* [pGB-DinB] cells remain sensitive to the large excess of DinB resulting from the combination of plasmid-borne expression and SOS induction.

### *trkA* and the *ssb* gene duplication are partially additive

The Δ*holD* mutant is rescued by a duplication of the *ssb* gene, and we proposed that this duplication allows a modification of the SSB-binding mode, from 65 nucleotides to 35 nucleotides bound by an SSB tetramer, which stabilizes HolD-less Pol III on DNA [16]. Low salts favor the (SSB)_35_ DNA binding mode *in vitro* ([37] and references therein), and to test whether the decrease in intracellular concentration of K^+^ in *trk* mutants suppressed the Δ*holD* mutant defects through a modification of the SSB binding mode, the *ssb* duplication was introduced in the Δ*holD* Δ*trkA* mutant. The *ssb* gene duplication showed an additive effect with Δ*trkA* mutation, allowing growth of the Δ*holD* Δ*trkA argE*::*ssb* triple mutant on M9 at 42°C (Fig 6). Rescue of the Δ*holD argE*::*ssb* mutants by Δ*trkA* was only efficient on 22 mM K^+^ and was not observed at low or high K^+^ concentrations, as expected for the role of intracellular K^+^ concentration in this phenomenon (S5 Fig). The additive effects of *ssb* gene duplication and *trkA* inactivation on Δ*holD* viability at 42°C suggests that Δ*trkA* does not act only by modifying the SSB binding mode. In fact, variations of temperature from 25°C to 37°C only modestly affect the SSB binding mode *in vitro* [38], 42°C was not tested), which disfavors the hypothesis that *in vivo* the *ssb* gene duplication promotes the (SSB)_35_ binding mode at 37°C and not at 42°C. If indeed in strains carrying the *ssb* duplication SSB binds DNA in the 35 base-pair mode at 42°C as at 37°C, the lethality of the *holD argE*::*ssb* mutant at 42°C results from a lack of stabilization of HolD-less Pol III by (SSB)_35_ at this temperature, and, in turn, the viability of the *holD argE*::*ssb trkA* triple mutant at 42°C suggests an effect of the *trkA* mutation on Pol III stability. However, we cannot exclude that *trk* mutations could promote a shift to the (SSB)_35_ at 30°C and 37°C on their own, and at 42°C in cells that carry the *ssb* gene duplication. Because these two modes of action are not exclusive and may have cumulative effects, the *trkA* mutation could stabilize Pol III both by enhancing directly its binding to DNA and by promoting the replication-favorable (SSB)_35_ binding mode, as discussed below.

## Discussion

In this study, we show that Δ*holD* and Δ*holC* mutants are viable when K^+^ import is affected. *trk* inactivation does not improve Δ*holD* viability by affecting SOS induction. We propose that decreasing K^+^ import improves electrostatic interactions between subunits in the defective Pol III HE and/or between Pol III and DNA, and may in addition stabilize Pol III on DNA indirectly by favoring the (SSB)_35_ binding mode, the combination of all these effects allowing chromosome replication by a defective Pol III HE and restoring viability.

### HolD plays a crucial role in strengthening DNA Pol III HE binding on DNA

The finding that decreasing the intracellular K^+^ concentration rescues the Δ*holD* mutant is in line with the idea that ψ (HolD) plays an important role *in vivo* in the stabilization of Pol III HE on DNA during replication. *In vitro*, ψ increases the affinity of τ for δδ’ in the core loading clamp pentameric complex, stabilizes the ATP-activated clamp loader complex conformational state, and increases the affinity of the clamp loader complex for the primer-template junction, thus favoring clamp loading [10–12]. Therefore ψ improves both protein-protein and protein-DNA interactions. The association of Pol III HE with DNA is intrinsically salt-dependent and strongly depends on the anion used for the reaction, with glutamate, the most physiological anion, protecting against high salt destabilizing effects [39]. Our results show that an intrinsically unstable Pol III HE can be stabilized by decreasing intracellular K^+^ concentration. However, our results argue that clamp loading during lagging-strand synthesis is still defective in the Δ*holD* Δ*trkA* mutant, since the SOS response remains highly induced, and RNA primers remain sensitive to an excess of RNaseH. Furthermore, the inactivation of *trk* improves the viability of the Δ*holC* mutant, although *in vitro* clamp-loader activity is mainly stimulated by ψ alone and the presence of χ only weakly affects the reaction [11]. Therefore, we propose that lowering K^+^ import restores the viability of the Δ*holD* mutant mainly by improving leading-strand replication, by limiting replication arrest or Pol III HE disassembly after arrest, or by facilitating replication restart.

A duplication of the *ssb* gene also rescues the *holD* mutant, and we hypothesized that this rescue results from the promotion of the (SSB)_35_ binding mode by a two-fold increase in intracellular SSB protein concentration. Because *in vitro* a shift from the (SSB)_65_ to the (SSB)_35_ binding mode can be promoted by increasing SSB concentration or decreasing salt concentration [37], it is conceivable that the 12–17% decrease in intracellular K^+^ concentration observed in *trk* mutants promotes the formation of (SSB)_35_, which, in turn, stabilizes the HolD-less Pol III on DNA. In conclusion, the *trkA* mutation could allow chromosome replication either by a direct effect on Pol III, if affecting potassium import increases electrostatic interactions between Pol III subunits and/or between Pol III and DNA, or indirectly, if it favors a shift to the SSB binding mode to (SSB)_35_. These two models are not exclusive and the *trk* mutation could affect Pol III stability on DNA by cumulative effects of a direct and an SSB-mediated stabilization. Furthermore, the observation that the difference in K^+^ intracellular concentration between wild-type and *trkA* mutants is only 12–17% supports the idea that viability of *holD trkA* mutant might result from a combination of potassium concentration effects.

Inhibition of SOS induction by a *lexAind* or a *recF* mutation restores full viability to Δ*holD* Δ*trkA* cells, including at high temperatures. This observation suggests that the remaining growth defects of Δ*holD* Δ*trkA* cells are caused by SOS-induced polymerases and that the combination of a more stable Pol III and less abundant Pol II and DinB competitors is sufficient to restore full viability. Altogether, we conclude that electrostatic interactions are crucial for replisome stability *in vivo* and can be improved beyond the wild-type level by decreasing K^+^ import.

### Effects of K^+^ on protein-nucleic acid interactions

K^+^ glutamate is the natural solute in *E*. *coli*, and is the major intracellular ionic osmolyte [20]. K^+^ intracellular concentration is regulated after hyper- or hypo-osmotic shocks by changes in the amount of water and by the action of several K^+^ import and efflux systems. In spite of a strong effect of salt concentration on protein-DNA interactions *in vitro*, K^+^ intracellular concentration can largely increase *in vivo* without affecting *lac* operator-repressor or phage λ RNA polymerase-promoter interactions [20]. Following a hyper-osmotic shock only some specific promoters are affected by the increased intracellular concentration of K^+^ glutamate: genes involved in osmotic protection are induced and ribosomal transcription is decreased [40]; effects of K^+^ transporters on the virulence of pathogenic bacteria were also reported [41, 42]. To account for the lack of effect of hyper-osmotic conditions on operator-repressor binding and promoter activity, it was proposed that increased intracellular K^+^ concentrations trigger a decrease in free cytoplasmic water, which enhances molecular crowding and thereby compensates for the destabilizing effect of the original K^+^ concentration increase [43–45]. Here the use of a mutant where the replication complex is intrinsically unstable on DNA allows us to show that protein-DNA interactions and possibly protein-protein interactions can be increased by lowering K^+^ import below the physiological wild-type level. We would like to propose the following hypotheses to account for the strong effect on viability in spite of a relatively weak K^+^ concentration decrease (i) either the replication machinery is highly sensitive to weak variations of intracellular K^+^ concentrations, for example because the effects on several replication proteins such as Pol III and SSB are cumulative as discussed above, (ii) or the 80 mM decrease in total K^+^ concentration that we observe in the *holD trk* mutants compared to wild-type affects only K^+^ ions free for exchange, leading to a 30% reduction of the potassium ions that affect DNA-protein interactions effectively, (iii) or *trk* and *kdp* mutations exert secondary effects on ions other than K^+^ that also control protein-DNA interactions. Strikingly, stabilization of Pol III HE on DNA, reflected by the Δ*holD* Δ*trkA* mutant viability, is particularly efficient in LB. The effects of LB are likely the result of a combination of multiple factors, including the presence of molecules such as glycine betaine and glutamate that stabilize protein-DNA complexes [39, 46]. However, only Δ*holD* mutants that lack the *trk* import system formed colonies on LB at 42°C, and not Δ*holD* mutants that lack SOS induction or carry an *ssb* gene duplication. Therefore, whatever the compounds that favor growth in LB, they are active in the *holD* mutant when combined with a limited K^+^ import.

Replication fork arrest is a recognized source of genome rearrangements in all organisms, and any replication defect can have severe consequences [47–50]. The identification of factors that improve replication fork stability in perturbed conditions is therefore crucial. Furthermore, theoretically mutations that affect protein-protein and protein-nucleic acid interactions in processes other than replication could also be suppressed by limiting K^+^ import. Our work underlines the influence of chemical intracellular composition on essential processes.

## Materials and Methods

### Strains, plasmids and media

Strains, plasmids and oligonucleotides used in this work are described in S1 Table. Genes were inactivated by recombineering as described in [51] using DY330 [52]. Mutations were transferred by P1 transduction. Antibiotics were used at the following concentrations: kanamycin (Kan) 50 μg/ml, chloramphenicol (Cm) 20 μg/ml, tetracycline (Tet) 15 μg/ml, ampicillin (Ap) 100 μg/ml, spectinomycin (Spc) 60 μg/ml. All minimum media used in this work contain 0.4% glucose, 0.2% casamino acids and 1 mg/L thiamine, except M9 tryptone medium which contains 0.4% tryptone instead of casamino acids. LB broth (Miller) is from Sigma, yeast extract, tryptone and casamino acids are from Difco. M9 is Na_2_HPO_4_ 42 mM, KH_2_PO_4_ 22 mM, NaCl 8.5 mM, NH_4_Cl 18.7 mM, MgSO_4_ 1 mM, CaCl_2_ 0.1 mM [53]; MMA is K_2_HPO_4_ 60.3 mM, KH_2_PO_4_ 33 mM, (NH_4_)_2_SO_4_ 7.6 mM, Na Citrate 1.7 mM, MgSO_4_ 1 mM [53]; MK115 is K_2_HPO_4_ 46 mM, KH_2_PO_4_ 23 mM, (NH_4_)_2_SO_4_ 8 mM, Na Citrate 1 mM, MgSO_4_ 0.4 mM, FeSO_4_ 6 μM [29]; MK0 is Na_2_HPO_4_ 46 mM, NaH_2_PO_4_ 23 mM, (NH_4_)_2_SO_4_ 8 mM, Mg SO_4_ 0.4 mM, FeSO_4_ 6 μM. Different amounts of MMA 2X or MK115 2X were added to MK0 for MK0.2, MK1 and MK22, to adjust to 0.2, 1 and 22 mM K^+^ respectively [29]. Strains containing pAM-holD were routinely grown in M9 containing 500 mM IPTG and 60 μg/ml spectinomycin at 37°C. pAM-holD (or pAM-holCD) were cured prior to each experiment by growing cells in the absence of IPTG, and plasmid-less colonies were isolated on M9 [16, 17]. We determined that less than 10% cells in the culture contain pAM-holD and less than 1% had acquired a suppressor mutation. Because of the high frequency of appearance of suppressor mutations, all new *holD* derivatives were constructed in the presence of pAM-holD. All mutations introduced by P1 transduction were verified by PCR, and all mutations constructed by recombineering were verified by PCR and sequencing. *lexAInd* and *recF* mutations were checked by measuring UV sensitivity.

### Viability measurement

For spot assays, plasmid-less colonies formed in three days on M9 at 30°C were suspended in M9 or MK0 salts. Serial 10-fold dilutions were performed and 5 μl of dilutions 10^−1^ to 10^−5^ were spotted on different media. Pictures of LB plates incubated at 37°C and 42°C were taken after 24 h incubation, for all *holD* mutants, pictures of LB plates incubated at 30°C and of minimum medium plates were taken after 2 days; for HolD^+^ strains for pictures of minimal medium plates incubated at 37°C were taken after 24 h incubation. All strains were tested at least three times independently. For growth curves, cultures of wild-type (JJC1392), *trkA* (JJC6800) and *holD trkA* [pAM-holD] (JJC6898) strains were grown overnight at 30°C in LB, M9, MK1, MK115 medium. Cells were diluted to O.D. 0.002 in the same medium and further grown at 37°C for 7 hours. This protocol was chosen because it allows overnight cultures and the subsequent growth curves to be performed in the same medium, without medium shift, and a direct comparison with the same protocol of viable (*holD trkA* in M9 and LB) and lethal (*holD trkA* in MK1 and MK115) growth conditions. The number of colony forming units per ml of culture (cfu/ml) was determined by plating appropriate dilutions on M9 and incubating plates at 30°C. The average percentage of plasmid-less cells, determined by plating appropriate dilutions on M9 with spectinomycin and IPTG, was independent of the medium, in average 74% after overnight propagation and 96% at the end of the growth curve. To verify that the *holD trkA* cultures did not acquire additional suppressor mutations during growth in M9, appropriate dilutions were also plated at 42°C, to check that cells were thermosensitive as expected.

### Genome sequencing

Chromosomal DNA was extracted using Sigma GenElute bacterial genomic DNA kit. 5 μg of DNA were used to generate a genomic library according to Illumina's protocol. The libraries and the sequencing were performed by the High-throughput Sequencing facility of the I2BC (http://www.i2bc.paris-saclay.fr/spip.php?article399&lang=en, CNRS, Gif-sur-Yvette, France). Genomic DNA libraries were made with the ‘Nextera DNA library preparation kit’ (Illumina) following the manufacturer’s recommendations. Library quality was assessed on an Agilent Bioanalyzer 2100, using an Agilent High Sensitivity DNA Kit (Agilent technologies). Libraries were pooled in equimolar proportions. Paired-end 2x250 bp reads were generated on an Illumina MiSeq instrument, using a MiSeq Reagent kit V2 (500 cycles) (Illumina), with an expected depth of 217X. Reads from mutant genome were aligned on the Escherichia coli K12 MG1655 genome using Illumina's package CASAVA 1.8.2. The point mutation and the small indels were detected also using Illumina's package CASAVA 1.8.2 and the large indels with profil visualisation and Blast (Basic Local Alignment Search Tool).

### Measurements of intracellular potassium concentration

Cells grown in M9 at 37°C until OD_650_ = 0.4 were cooled in ice, harvested by centrifugation, and washed three times in cold hyper-tonic medium: 1.mM Tris-Cl (pH 8), 1 mM MgSO_4_, and 0.4 M glucose [31]. Pellets were dried overnight at 56°C. Dry pellets were weighted, digested in 2 ml of HNO_3_ >68% (20 min at 80°C and 1h at 120°C), diluted 50 fold in H_2_O, and K^+^ was measured by flame spectrophotometry using a Varian AA240FS spectrophotometer and a range of 0.1 to 5 mg/L K^+^ standard solutions.

### β-galactosidase assays

β-galactosidase assays for measures of SOS induction were performed as described previously [16, 53]. Since isolated JJC6545 and JJC7058 colonies could not be cultivated owing to the growth advantage of suppressor mutations, pAM-*holD* containing clones were grown overnight in M9 lacking IPTG and diluted 50 fold in M9 for the experiment. Cultures were tested for the loss of pAM-*holD* and for containing less than 1% suppressor mutations.

## Supporting Information

S1 Fig *trkA* 84 pb deletion in JJC6377. Top and bottom lines show the wild-type *trkA* gene in the region of the deletion. Middle line is the sequence of *trkA*^Δ84^. The 9 pb microhomology is in bold, the upstream sequence is in blue and the downstream sequence in purple. Numbers refer to the position in *trkA*, when the A in the ATG is numbered 1.
(PDF)

S2 Fig *holD trk* mutants are sensitive to high and low potassium concentrations. Serial dilutions used in Fig 2 were plated on MM containing different concentrations of K+ and incubated at the indicated temperature.
(PDF)

S3 Fig Isolated colonies were obtained by plating on M9 appropriate dilutions of cultures propagated for 8 hours in the absence of IPTG and incubating plates for 3 days at 30°. Serial 10-fold dilutions were made and 5μl drops of each dilution were spotted on minimal medium M9, LB, or MK plates containing the indicated potassium concentration. A. Δ*kup* mutation does not affect the growth of Δ*holD* or Δ*holD* Δt*rkA* mutants. Plates were incubated for two days at the indicated temperature. *holD*, JJC6869; *holD kup*, JJC7001; *holD trkA*, JJC6898; *holD trkA kup*, JJC7002. B. The presence of casaminoacids in minimal medium does not affect viability In this work all minimal medium plates contain 0.2% casaminoacids. The presence of casaminoacids increased growth rates but did not affect viability. Strains are as in Fig 4: wild-type, JJC1392; *holD trkE sup*, JJC6389; strains used to get plasmid-less colonies: *holD*, JJC6869; *holD trkA*, JJC6898; *holD trkA*^Δ84^, JJC6969. Plates were incubated at 42°C or 37°C for two days or at 30°C for three days. Note that in this particular experiment the *holD* mutant colony that was used contained a higher than usual sub-population of suppressors allowing growth at 30°C (compare with S3A Fig). Such jackpots of suppressors were observed in less than 10% of plasmid-less *holD* colonies.
(PDF)

S4 Fig Rescue of the *holC* and *holC holD* mutants by *trkA* inactivation depends on potassium concentration. Serial dilutions used in Fig 5 were plated on MK medium containing different concentrations of K^+^ and incubated at the indicated temperature.
(PDF)

S5 Fig Rescue of the *holD lexAind* and *holD argE*::*ssb* mutants by *trkA* inactivation depends on potassium concentration. Serial dilutions used in Fig 6B were plated on MK medium containing different concentrations of K^+^ and incubated at the indicated temperature.
(PDF)

S1 Table Strains, plasmids and oligonucleotides. (PDF)
