# Supplementary material for: Mutations Affecting Potassium Import Restore the Viability of the Escherichia coli DNA Polymerase III holD Mutant
Source: PLoS Genet. 2016 Jun 9;12(6):e1006114. doi: 10.1371/journal.pgen.1006114 (PMC4900610; doi:10.1371/journal.pgen.1006114)
Supplement: S1 Fig — Top and bottom lines show the wild-type trkA gene in the region of the deletion. Middle line is the sequence of trkAΔ84. The 9 pb microhomology is in bold, the upstream sequence is in blue and the downstream sequence in purple. Numbers refer to the position in trkA, when the A in the ATG is numbered 1. (PDF) [file pgen.1006114.s001.pdf]

Figure S1. *trkA* 84 pb deletion in JJC6377.

513 **521**

TGGTAATGCACT**TTCGACCAT**GCGCGAACATA

trkAΔ84 TGGTAATGCACT**TTCGACCAT**TGTTGAAGCTG

TCGTCCGCAAGG**TTCGACCAT**TGTTGAAGCTG

597 **605**
