# Supplementary figures and images for: Mutations Affecting Potassium Import Restore the Viability of the Escherichia coli DNA Polymerase III holD Mutant
Source: PLoS Genet. 2016 Jun 9;12(6):e1006114. doi: 10.1371/journal.pgen.1006114 (PMC4900610; doi:10.1371/journal.pgen.1006114)

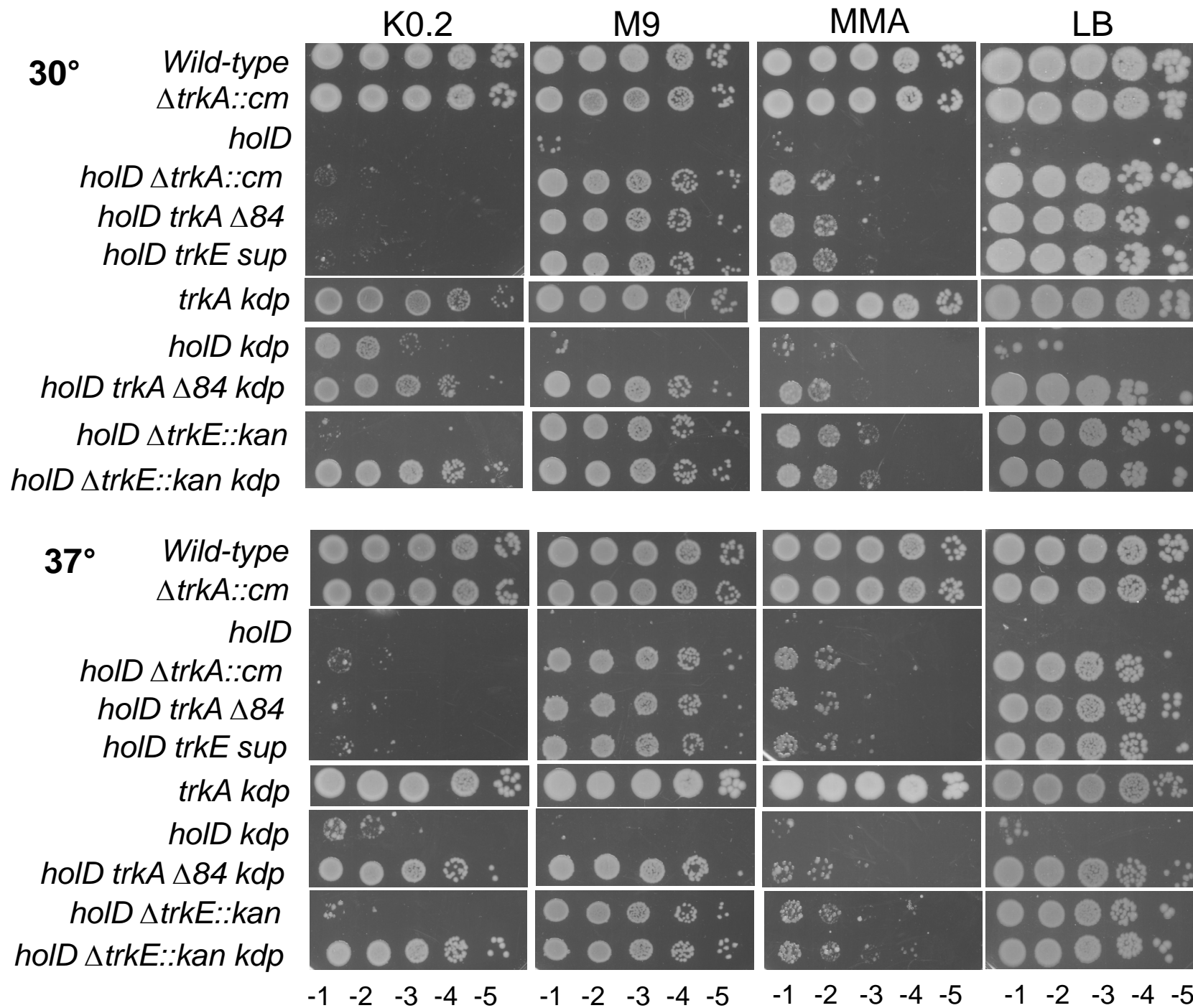

Supplement: S2 Fig — Serial dilutions used in Fig 2 were plated on MM containing different concentrations of K+ and incubated at the indicated temperature. (PDF) [file pgen.1006114.s002.pdf]

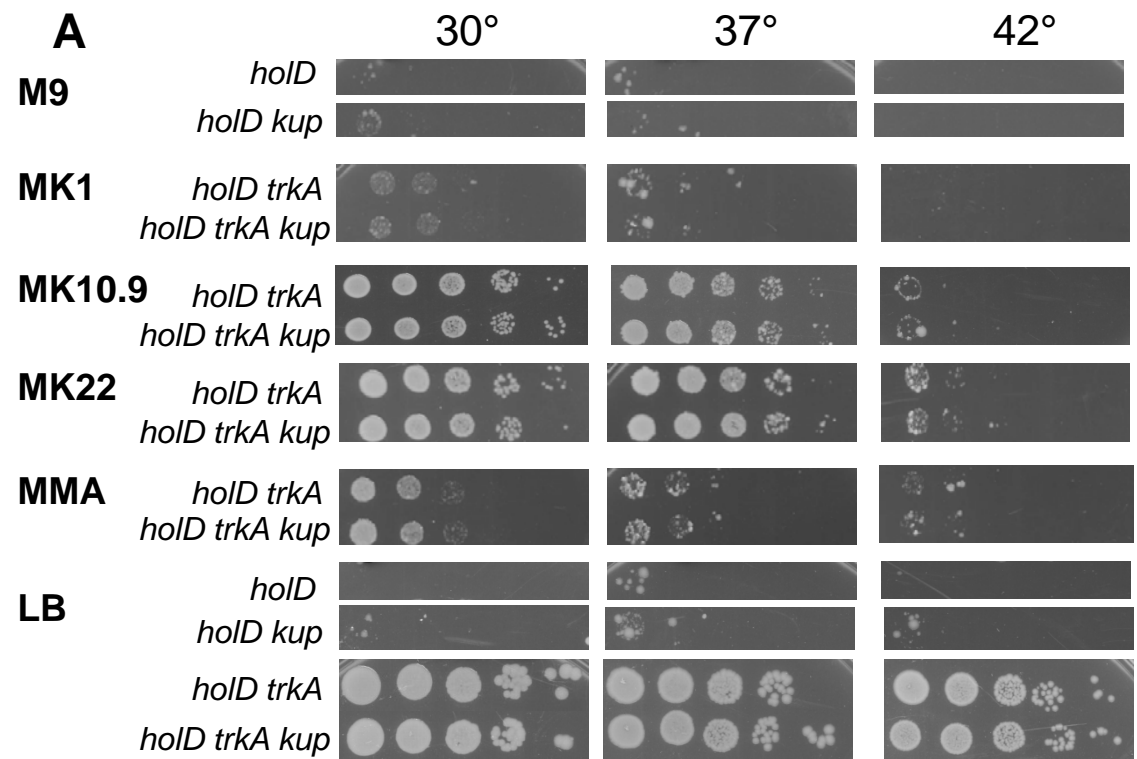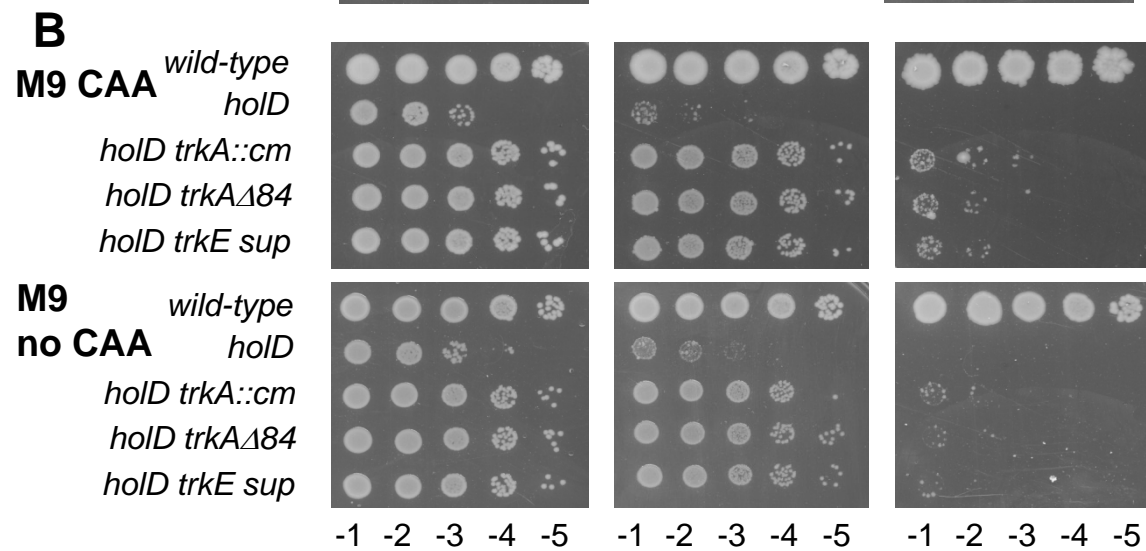

Supplement: S3 Fig — Serial 10-fold dilutions were made and 5μl drops of each dilution were spotted on minimal medium M9, LB, or MK plates containing the indicated potassium concentration. A. Δkup mutation does not affect the growth of ΔholD or ΔholD ΔtrkA mutants. Plates were incubated for two days at the indicated temperature. holD, JJC6869; holD kup, JJC7001; holD trkA, JJC6898; holD trkA kup, JJC7002. B. The presence of casaminoacids in minimal medium does not affect viability In this work all minimal medium plates contain 0.2% casaminoacids. The presence of casaminoacids increased growth rates but did not affect viability. Strains are as in Fig 4: wild-type, JJC1392; holD trkE sup, JJC6389; strains used to get plasmid-less colonies: holD, JJC6869; holD trkA, JJC6898; holD trkAΔ84, JJC6969. Plates were incubated at 42°C or 37°C for two days or at 30°C for three days. Note that in this particular experiment the holD mutant colony that was used contained a higher than usual sub-population of suppressors allowing growth at 30°C (compare with S3A Fig). Such jackpots of suppressors were observed in less than 10% of plasmid-less holD colonies. (PDF) [file pgen.1006114.s003.pdf]

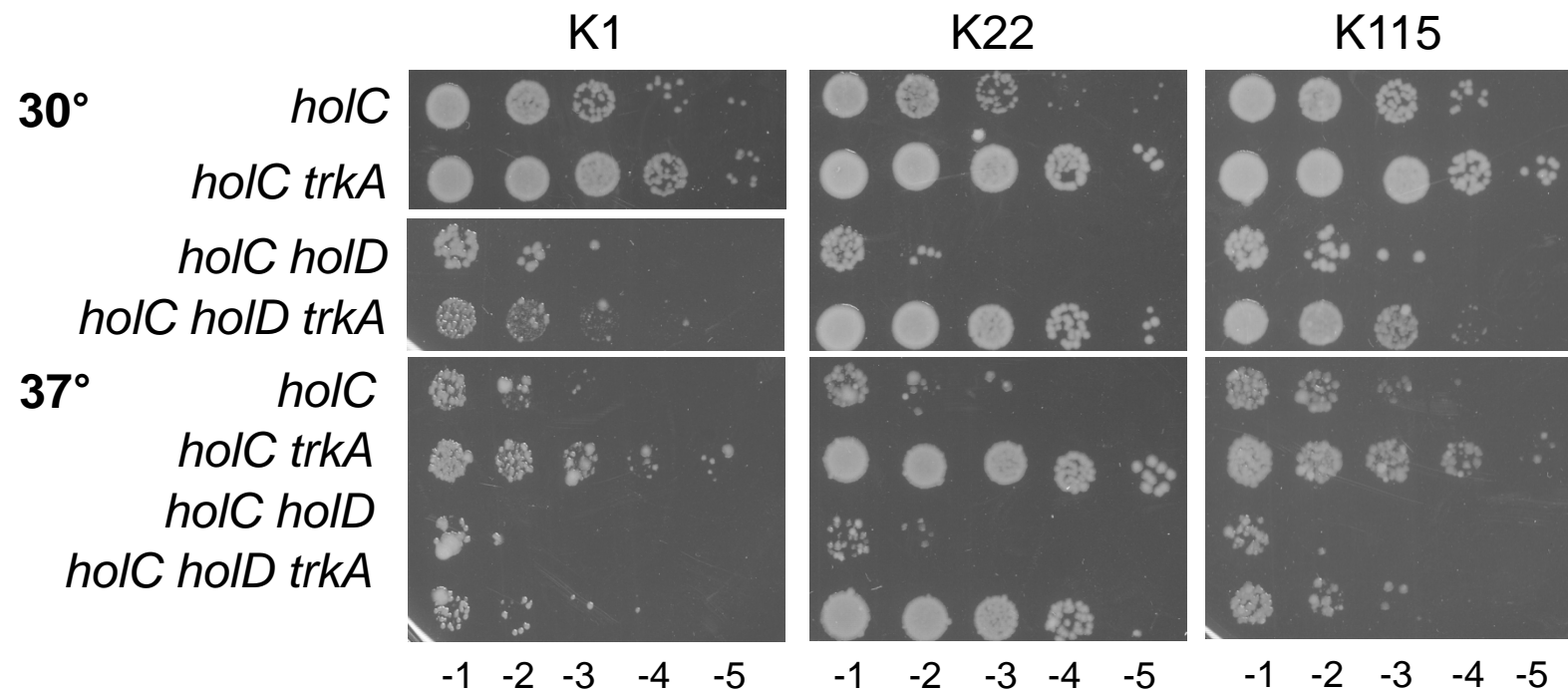

Supplement: S4 Fig — Serial dilutions used in Fig 5 were plated on MK medium containing different concentrations of K+ and incubated at the indicated temperature. (PDF) [file pgen.1006114.s004.pdf]

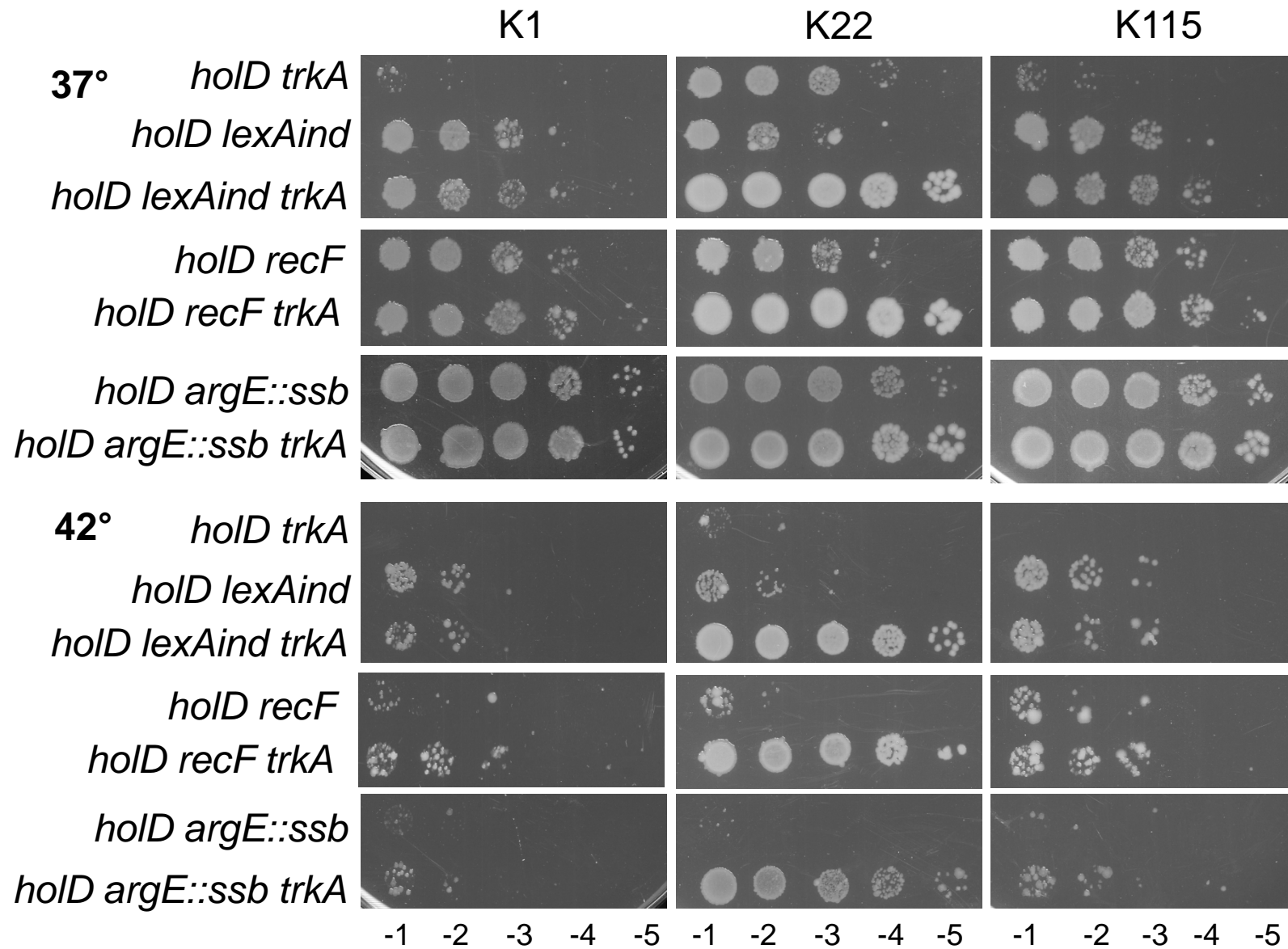

Supplement: S5 Fig — Serial dilutions used in Fig 6B were plated on MK medium containing different concentrations of K+ and incubated at the indicated temperature. (PDF) [file pgen.1006114.s005.pdf]
