# Supplementary material for: Mutations Affecting Potassium Import Restore the Viability of the Escherichia coli DNA Polymerase III holD Mutant
Source: PLoS Genet. 2016 Jun 9;12(6):e1006114. doi: 10.1371/journal.pgen.1006114 (PMC4900610; doi:10.1371/journal.pgen.1006114)
Supplement: S1 Table — (PDF) [file pgen.1006114.s006.pdf]

Table S1 Strains

Unless indicated, all strains derive from MG1655.

Name	Relevant genotype	Construction or reference
CAG12071	*zhd*3082::Tn*10*	[1]
DY330	W3110 Δ*lacU169 gal490* λCI^{857} Δ(*cro-bio*A)	[2]
JW5609	Δ*kup*::kan	Keio collection
JJC443	*lexAind3 mal*::Tn*10*	Laboratory collection
JJC450	AB1157 *recF400*::Tn*5*	Laboratory collection
JJC1392	Wild-type MG1655	C. Gross
JJC1548	*sfiA*::MudAp*lacZ*	Laboratory collection
JJC1549	AB1157 Δ*holD*::kan [pAM-holD]	[3]
JJC2062	AB1157 *recF400*::Tn*5 zid501*::Tn*10*	Laboratory collection
JJC3523	Δ*lacZ*	[4]
JJC5954	*argE*::*ssb*-kan	[4]
JJC6356	Δ*lacZ* Δ*holD*::kan [pAM-holD]	[4]
JJC6363	Δ*lacZ* Δ*holD*::kan *sfiA*::MudAp*lacZ* [pAM-holD]	[4]
JJC6377	Δ*lacZ* Δ*holD*::kan *trkA*$^{\Delta 84}$ ~500 kb duplication (3 648 177-4 166 720)	Spontaneous JJC6356 suppressor, selected at 37°C
JJC6389	Δ*lacZ* Δ*holD*::kan *trkE*^{Q255P}	Spontaneous JJC6363 suppressor, selected at 30°C
JJC6394	Δ*lacZ* Δ*holD*::FRT *argE*::*ssb*-kan *sfiA*::MudAp*lacZ* [pAM-holD]	[4]
JJC6420	Δ*lacZ* Δ*holD*::kan *lexAind3 mal*::Tn*10 sfiA*::MudAp*lacZ* [pAM-holD]	[4]

JJC6436	Δ*lacZ* Δ*holD*::kan *trkA*$^{\Delta 84}$ *zhd*3082::Tn*10* (*smg*)	JJC6377 * P1 CAG12071
JJC6478	Δ*lacZ sfiA*::MudAp*lacZ*	JJC3523 * P1 JJC1548
JJC6521	Δ*sfiA*::cm	Laboratory collection
JJC6534	Δ*sfiA*::FRT	JJC6521 cm^{S} by FRT recombination
JJC6535	Δ*lacZ sfiA*::MudAp*lacZ* [pAM-holD]	JJC6478 transformed with pAM-holD
JJC6545	Δ*lacZ sfiA*::MudAp*lacZ* Δ*holD*::kan [pAM-holD]	JJC6535 * P1 JJC1489
JJC6654	Δ*lacZ* Δ*holD*::kan *trkA*$^{\Delta 84}$ [pAM-holD]	JJC6377 transformed with pAM-holD
JJC6659	DY330 Δ*trkA* :: Cm	DY330 transformed with the PCR fragment Δ*trkA* :: Cm
JJC6669	Δ*lacZ* Δ*holD*::kan *trkA*$^{\Delta 84}$ *zhd*3082::Tn*10* [pAM-holD]	JJC6356 * P1 JJC6436
JJC6670	Δ*lacZ* Δ*holD*::kan *trkA*$^{+}$ *zhd*3082::Tn*10* [pAM-holD]	JJC6356 * P1 JJC6436
JJC6672	Δ*lacZ* Δ*holD*::kan *sfiA*::MudAp*lacZ* *trkE*^{Q255P} [pAM-holD]	JJC6389 transformed with pAM-holD
JJC6674	Δ*holD*::kan Δ*lacZ trkA*$^{\Delta 84}$ *zhd*3082::Tn*10* [pAM-holD]	JJC6654 * P1 CAG12071
JJC6682	Δ*lacZ* Δ*holD*::kan Δ*trkA*::cm [pAM-holD]	JJC6356 * P1 JJC6659
JJC6683	Δ*lacZ* Δ*trkA*::cm	JJC3523 * P1 JJC6659
JJC6720	DY330 Δ*kdp*::cm	DY330 transformed with the PCR fragment Δ*kdp*:: cm
JJC6726	Δ*sfiA*::FRT [pAM-holD]	JJC6534 transformed with pAM-holD
JJC6729	Δ*sfiA*::FRT Δ*holD*::kan [pAM-holD]	JJC6726 * P1 JJC1489
JJC6740	Δ*sfiA*::FRT [pAM-holCD]	JJC6534 transformed with pAM-holCD
JJC6747	Δ*sfiA*::FRT Δ*holD*::kan [pAM-holCD]	JJC6740 * P1 JJC1489
JJC6748	Δ*sfiA*::FRT Δ*holC*102::cmR *zig*2086::kanR [pAM holCD]	JJC6740 * P1 JJC1098

JJC6750	Δ*lacZ* Δ*holD*::kan Δ*kdp*::cm [pAM-holD]	JJC6356 * P1 JJC6720
JJC6767	Δ*sfiA*::FRT Δ*holD*::FRT [pAM-holCD]	JJC6747 cm^S by FRT recombination
JJC6774	Δ*sfiA*::FRT Δ*holD*::FRT Δ*holC*102::cmR *zig*2086::kan [pAM-holCD]	JJC6767 * P1 JJC1098
JJC6800	Δ*sfiA*::FRT Δ*trkA*::cm	JJC6534 * P1 JJC6659
JJC6801	Δ*sfiA*::FRT Δ*holD*::kan Δ*trkA*::cm [pAM holCD]	JJC6747 * P1 JJC6659
JJC6819	Δ*lacZ* Δ*holD*::kan $trkA^{\Delta 84}$ *zhd*3082::Tn*10* Δ*kdp*::cm [pAM holD]	JJC6669 * P1 JJC6720
JJC6826	Δ*sfiA*::FRT Δ*holC*102::cm *zig*2086::kan $trkA^+$ *zhd*3082::Tn*10* [pAM holCD]	JJC6748 * P1 JJC6436
JJC6827	Δ*sfiA*::FRT Δ*holC*102::cm *zig*2086::kan $trkA^{\Delta 84}$ *zhd*3082::Tn*10* [pAM holCD]	JJC6748 * P1 JJC6436
JJC6828	Δ*sfiA*::FRT Δ*holD*::FRT Δ*holC*102::cm *zig*2086::kan $trkA^{\Delta 84}$ *zhd*3082::Tn*10* [pAM holCD]	JJC6774 * P1 JJC6436
JJC6869	Δ*sfiA*::FRT Δ*holD*::FRT [pAM-holD]	JJC6729 kan^S by FRT recombination
JJC6881	Δ*sfiA* FRT Δ*holD*::FRT Δ*trkA*::cm [pAM holCD]	JJC6767 * P1 JJC6659
JJC6896	Δ*lacZ* *sfiA*::MudAp*lacZ* $trkA^+$ *zhd*3082::Tn*10*	JJC6478 * P1 JJC 6436
JJC6897	Δ*lacZ* *sfiA*::MudAp*lacZ* $trkA^{\Delta 84}$ *zhd*3082::Tn*10*	JJC6478 * P1 JJC6436
JJC6898	Δ*sfiA*::FRT Δ*holD* FRT Δ*trkA*::cm [pAM holD]	JJC6869 * P1 JJC6659
JJC6899	Δ*sfiA*::FRT Δ*holD*::FRT Δ*holC*102::cmR zig2086::*kan*R *trkA*+ *zhd*::Tn*10* [pAM holCD]	JJC6774 * P1 JJC6436
JJC6904	Δ*sfiA*::FRT *lexA*Def71::Tn*5*	Laboratory collection
JJC6910	Δ*holD*::kan Δ*trkA*::Cm	JJC6801 cured of pAM-holD
JJC6927	Δ*sfiA*::FRT Δ*holD* FRT Δ*trkA*::cm	JJC6898 cured of pAM-holD
JJC6928	Δ*sfiA*::FRT Δ*holD*::FRT Δ*holC*102::cmR *zig*2086::kanR $trkA^{\Delta 84}$ *zhd*3082::Tn*10*	JJC6828 cured of pAM-holD

JJC6929	Δ*sfiA*::FRT Δ*holD*::FRT Δ*holC102*::cmR *zig2086*::kanR *trkA*$^{+}$ *zhd*3082::Tn*10*	JJC6899 cured of pAM-holD
JJC6968	Δ*lacZ sfiA*::MudAp*lacZ* *trkA*$^{+}$ *zhd*::Tn*10* Δ*holD*::kan [pAM-holD]	JJC6545 * P1 JJC6436
JJC6969	Δ*lacZ sfi*A::MudAp*lacZ* *trkA*$^{\Delta 84}$ *zhd*3082::Tn*10* Δ*holD*::kan [pAM-holD]	JJC6545 * P1 JJC6436
JJC7000	Δ*sfiA*::FRT Δ*holD* FRT Δ*trkA*::cm *argE*::*ssb*-kan [pAM-holD]	JJC6898 * P1 JJC5954
JJC7001	Δ*sfiA*::FRT Δ*holD*::FRT Δ*kup*::kan [pAM-holD]	JJC6869 * P1 JJC6998
JJC7002	Δ*sfiA*::FRT Δ*holD*::FRT Δ*trkA*::cm Δ*kup*::kan [pAM-holD]	JJC6898 * P1 JJC6998
JJC7004	Δ*sfiA*::FRT Δ*holD* FRT Δ*trkA*::cm *lexAind3 mal*::Tn*10*	Plasmid-less JJC7008
JJC7008	Δ*sfiA*::FRT Δ*holD* FRT Δ*trkA*::cm *lexAind3 mal*::Tn*10* [pAM-holD]	JJC6898 * P1 JJC443
JJC7011	*trkA*$^{\Delta 84}$ *zhd*3082::Tn*10*	JJC 1392 * P1 JJC6436
JJC7021	Δ*trkE*::kan (originally called Δ*sapD*::kan)	JW1284 Keio collection
JJC7030	*trkA*$^{\Delta 84}$ *zhd*3082::Tn*10* Δ*kdp*::cm	JJC7011 * P1 JJC6720
JJC7057	Δ*sfiA*::FRT Δ*holD* FRT Δ*trkA*::cm *recF400*::Tn*5* [pAM holD]	JJC6898 * P1 JJC450
JJC7058	Δ*lacZ sfiA*::MudAp*lacZ* Δ*holD*::kan [pAM-holD] *recF400*::Tn*5* *zid501*::Tn*10*	JJC6545 * P1 JJC2062
JJC7063	Δ*sfiA*::FRT Δ*holD* FRT Δ*trkA*::cm *recF400*::Tn*5*	Plasmid-less JJC7057
JJC7173	Δ*sfiA*::FRT Δ*holD* FRT Δ*trkE*::kan [pAM holD]	JJC6869 * P1 JJC7021
JJC7223	Δ*sfiA*::FRT Δ*holD* FRT Δ*trkE*::kan Δ*kdp*::cm [pAM holD]	JJC7173 * P1 JJC6720
JJC7237	Δ*sfiA*::FRT Δ*trkA*::FRT	JJC6683 kan^{S} by FRT recombination
JJC7251	Δ*sfiA*::FRT Δ*trkA*::FRT [pAM-holD]	JJC7237 transformed with pAM-holD
JJC7267	Δ*sfiA*::FRT Δ*trkA*::FRT [pAM-holD] [pACYC184]	JJC7251 transformed with pACYC184
JJC7268	Δ*sfiA*::FRT Δ*trkA*::FRT [pAM-holD] [pEM001]	JJC7251 transformed with pEM001
**Plasmids**		

pAM34	pBR322-derived plasmid where the RNA primer original promoter was replaced by the *lac* promoter; carries Ap^R, $Spec^R$ and *lacI* genes	Gil and Bouché
pAM-*holD*	pAM34 carrying the *holD* gene cloned in *Eco*RI; Ap^R, $Spec^R$	[3]
pAM-*holC*	pAM34 carrying the *holC* gene cloned in *Bam*HI-*Hind*III; Ap^R	[4]
pAM-*holCD*	pAM-*holD* carrying the *holC* gene cloned in *Bsa*BI-*Xba*I; Ap^R, $Spec^R$	[4]
pGB-*dinB*	pGB2-derived plasmid carrying the wild-type *dinB* gene	[5]
pGB-*dinB*ΔC5	pGB2-derived plasmid carrying the *dinB* gene deleted for the 5 terminal amino acids	[5]
pEM001	pACYC184-derived plasmid with *rnh* gene	[6]
**Oligonucleotides**		
Number	Sequence	Use
#110	CGCGGGTGGGCTTGATAAAC	check *argE*::*ssb*
#153	TCGAGATTTACCGCGCTCTG	check *argE*::*ssb*
#146	TCTTCGGCCAGTAGTAAATCAGC	check *holD*
#147	GTAATTGCGGCGAATCGTCG	check *holD*
#420	CGCGCATGGCGCTTACCGCTGG	check *holC*
#421	CGAACAGCCGCTTTACGAGCAC	check *holC*
#424	AGCCATTTTCCTGCCAGGA	Check *trkA*
#451	CAGGAACACCAGAGCAACC	Check *trkA*

#483	GTTGGTTAACCTCCTTGGC	Check *trkE*
#484	AAACCAGCCGGTCCGGTA	Check *trkE*
#508	ATG AAA ATT ATC ATT CTG GGT GCC GGC CAG GTT GGC GGC ACA CTG GCG GAG TGT AGG CTG GAG CTG CTT C	Construction Δ*trkA*::cm
#509	ATC CTT GAT AAT CAA AGG GAA ATA ATA AGG CGT CAT TAG ACG CCT TAT TAC ACT TAA CGG CTG ACA TGG G	Construction Δ*trkA*::cm
#514	TCA TGG CTT TTG CCA TTT TTA TAC TTT TTT TAC ACC CCG CCC GCA GAT TTG TGT AGG CTG GAG CTG CTT C	Construction Δ*kdp*::cm
#515	CGA TGC TAA TAC AGG TGG TCA GCA GAC TGC CGA TCC AGA CGA TAA ACA TCC ACT TAA CGG CTG ACA TGG G	Construction Δ*kdp*::cm
#518	TTTACTACTCATCCGACCAC	Check *kdp*
#519	GAACAGTACGGTGATCCACA	Check *kdp*
#557	TAATCGTGCATACTGTGCGC	Check *kup*
#558	AAACCATCGTGACGTTACGG	Check *kup*

1. Nichols BP, Shafiq O, Meiners V (1998) Sequence analysis of Tn10 insertion sites in a collection of Escherichia coli strains used for genetic mapping and strain construction. J Bacteriol 180: 6408-6411.
2. Yu D, Ellis HM, Lee EC, Jenkins NA, Copeland NG, et al. (2000) An efficient recombination system for chromosome engineering in Escherichia coli. Proc Natl Acad Sci U S A 97: 5978-5983.
3. Viguera E, Petranovic M, Zahradka D, Germain K, Ehrlich DS, et al. (2003) Lethality of bypass polymerases in Escherichia coli cells with a defective clamp loader complex of DNA polymerase III. Mol Microbiol 50: 193-204.
4. Duigou S, Silvain M, Viguera E, Michel B (2014) ssb gene duplication restores the viability of DeltaholC and DeltaholD Escherichia coli mutants. PLoS Genet 10: e1004719.
5. Lenne-Samuel N, Wagner J, Etienne H, Fuchs RP (2002) The processivity factor beta controls DNA polymerase IV traffic during spontaneous mutagenesis and translesion synthesis in vivo. EMBO Rep 3: 45-49.

6. Masse E, Phoenix P, Drolet M (1997) DNA topoisomerases regulate R-loop formation during transcription of the rrnB operon in Escherichia coli. J Biol Chem 272: 12816-12823.
